# Supplementary material for: Elevated polygenic burden for autism is associated with differential DNA methylation at birth
Source: Genome Med. 2018 Mar 28;10:19. doi: 10.1186/s13073-018-0527-4 (PMC5872584; doi:10.1186/s13073-018-0527-4)

Figure S1. Histogram of days between birth and neonatal blood spot sampling for the MINERvA cohort.

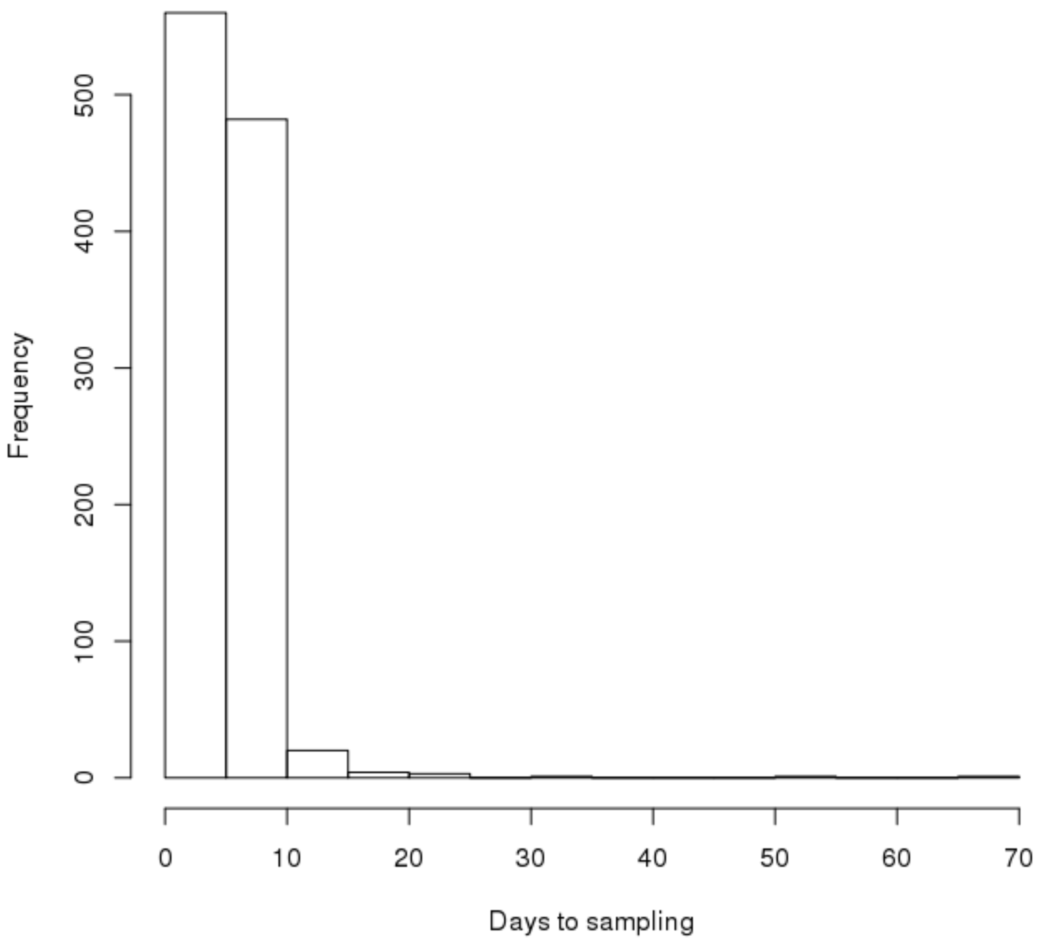

Figure S2. Histogram of gestational age predicted from DNA methylation data in the MINERvA cohort using an algorithm described by Knight et al (2016).

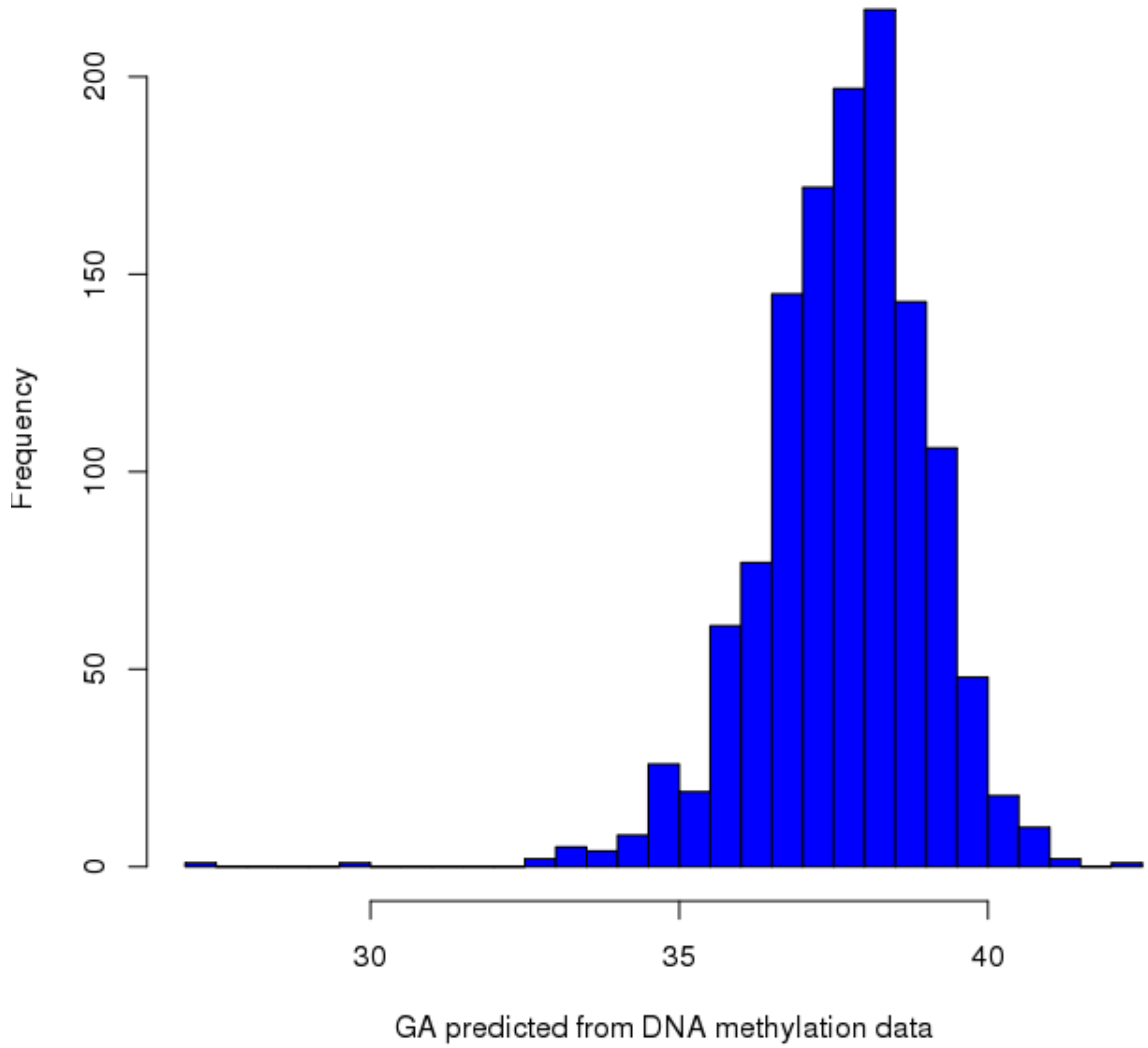

Knight, A.K., Craig, J.M., Theda, C., Bækvad-Hansen, M., Bybjerg-Grauholm, J., Hansen, C.S., Hollegaard, M.V., Hougaard, D.M., Mortensen, P.B., Weinsheimer, S.M., et al. (2016). An epigenetic clock for gestational age at birth based on blood methylation data. Genome Biol 17, 206.

Figure S3. Histogram of age predicted from DNA methylation data in the MINERvA cohort using the online Epigenetic Clock software (Horvath et al, 2013).

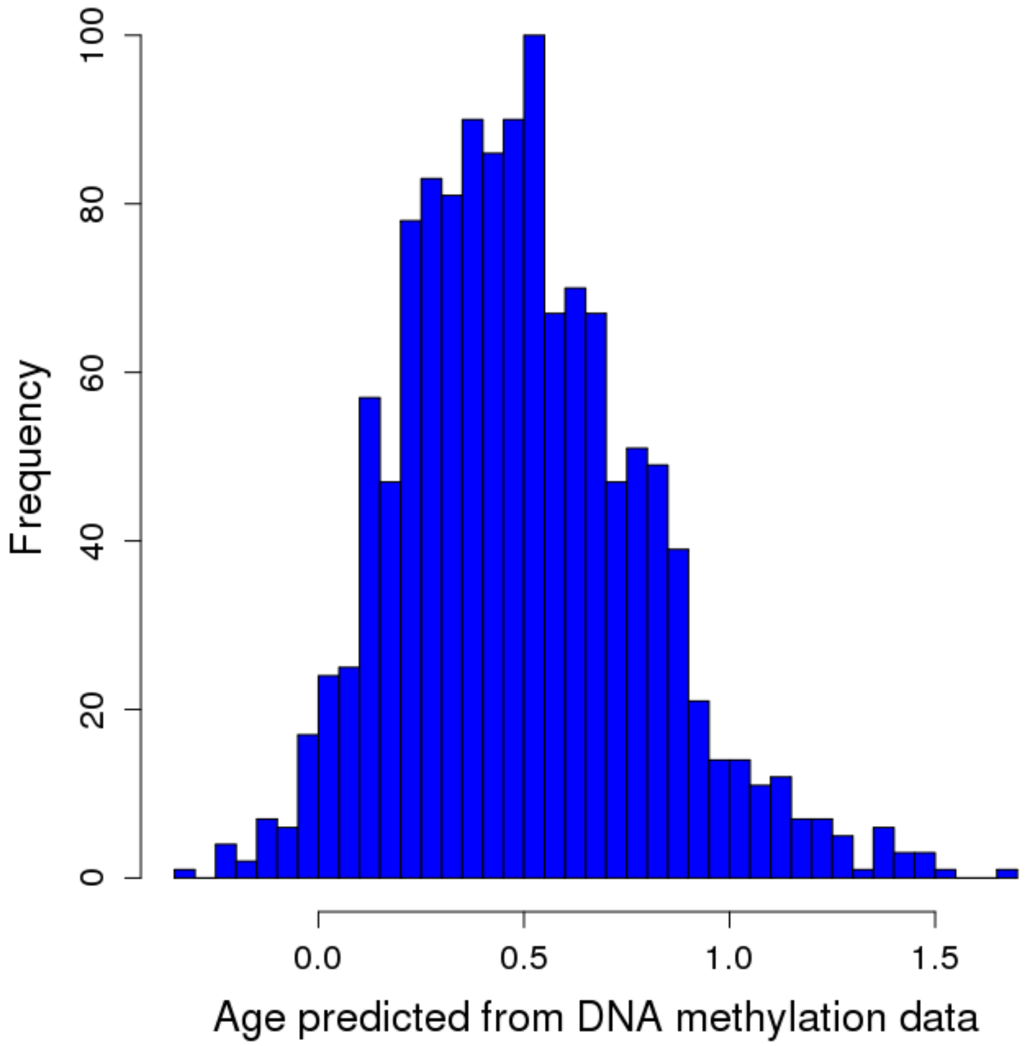

Horvath, S. (2013). DNA methylation age of human tissues and cell types. Genome Biol 14, R115.

Figure S4. Age estimates derived from neonatal blood DNA methylation data accurately reflect actual age. Scatterplots of derived age (top row) and gestational age (bottom row) estimated from DNA methylation using two different clock algorithms (Horvath et al, 2013, Knight et al, 2016) against gestational age in weeks (first column), days between birth and sampling (middle column) and gestational age corrected for days between birth and sampling (third column) for individual samples profiled in the Minerva cohort (n = 1,263).

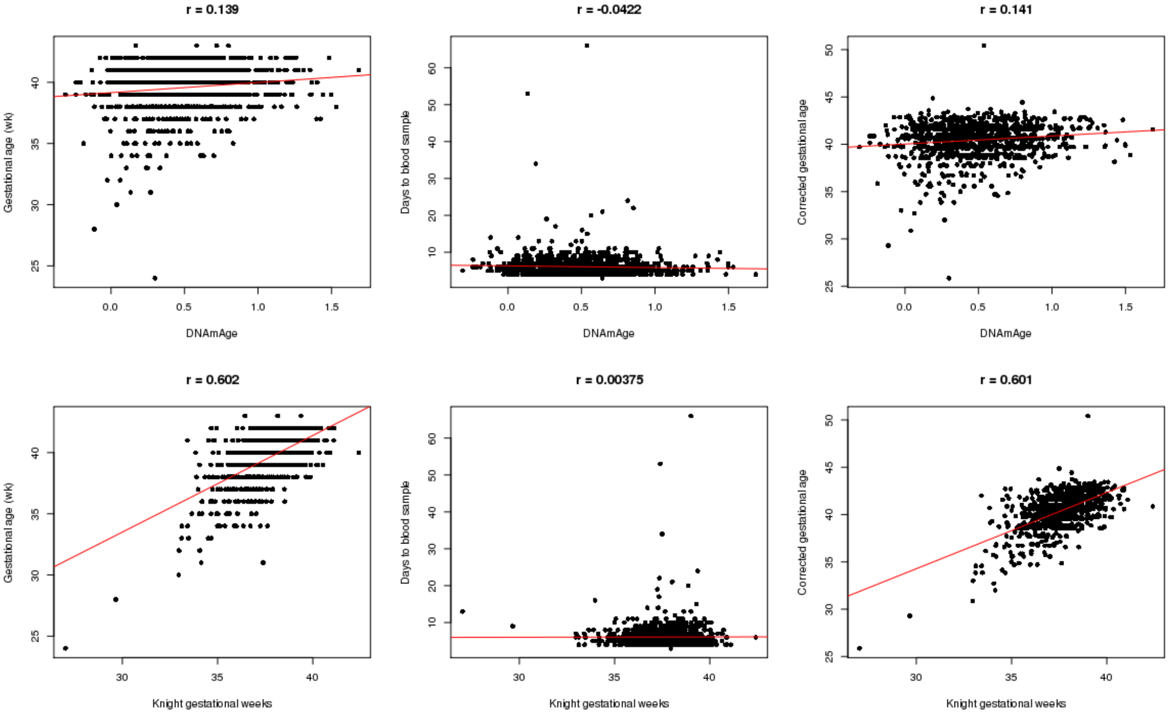

Knight, A.K., Craig, J.M., Theda, C., Bækvad-Hansen, M., Bybjerg-Grauholm, J., Hansen, C.S., Hollegaard, M.V., Hougaard, D.M., Mortensen, P.B., Weinsheimer, S.M., et al. (2016). An epigenetic clock for gestational age at birth based on blood methylation data. Genome Biol 17, 206.
Horvath, S. (2013). DNA methylation age of human tissues and cell types. Genome Biol 14, R115.

Figure S5. QQ plot of P-values from the epigenome-wide association study (EWAS) of autism case-control status in neonatal blood spots (n = 1,263).

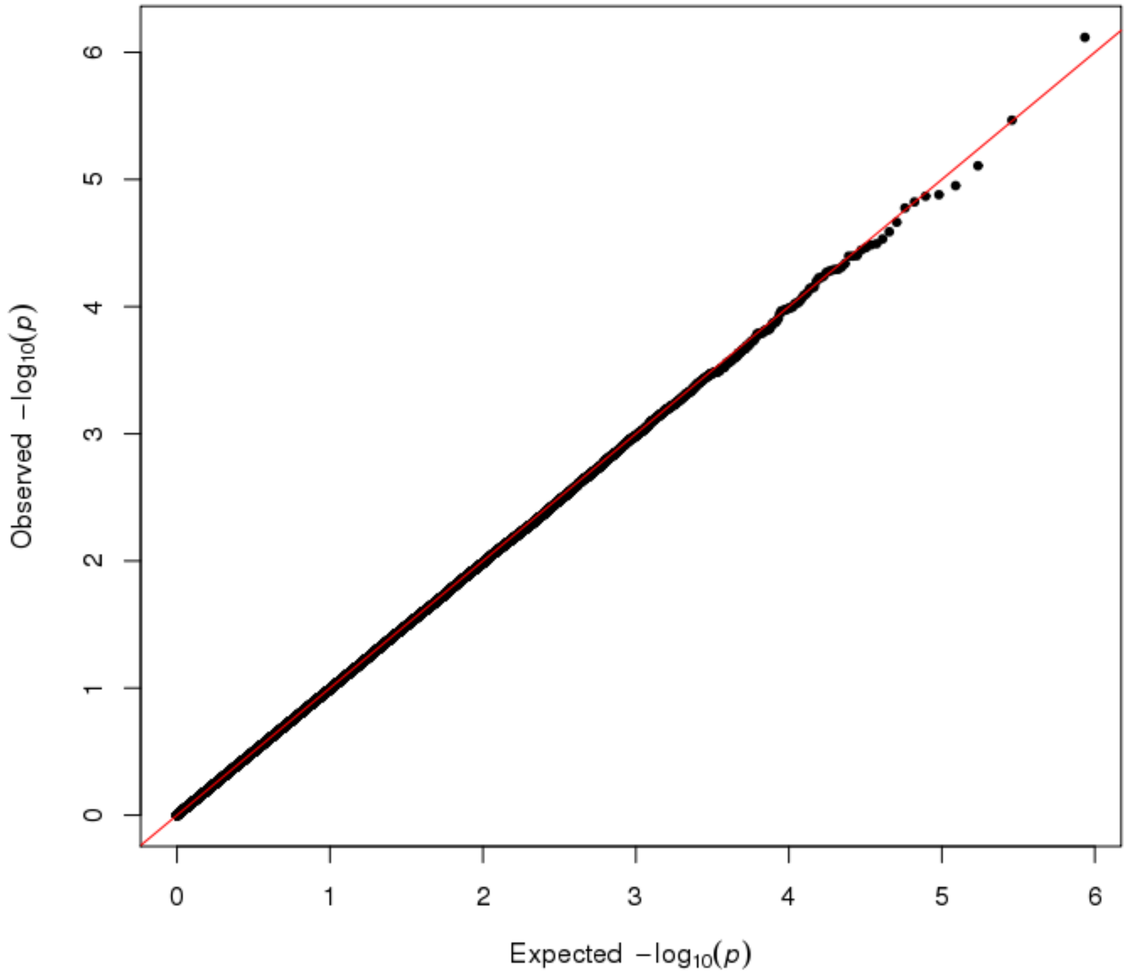

Figure S6. Manhattan plot of P-values from the autism case-control EWAS in neonatal blood spots (n = 1,263). The red horizontal line indicates the experiment-wide significance threshold ($P < 1x10^{-7}$). The blue horizontal line indicates a suggestive significance threshold ($P < 5x10^{-5}$).

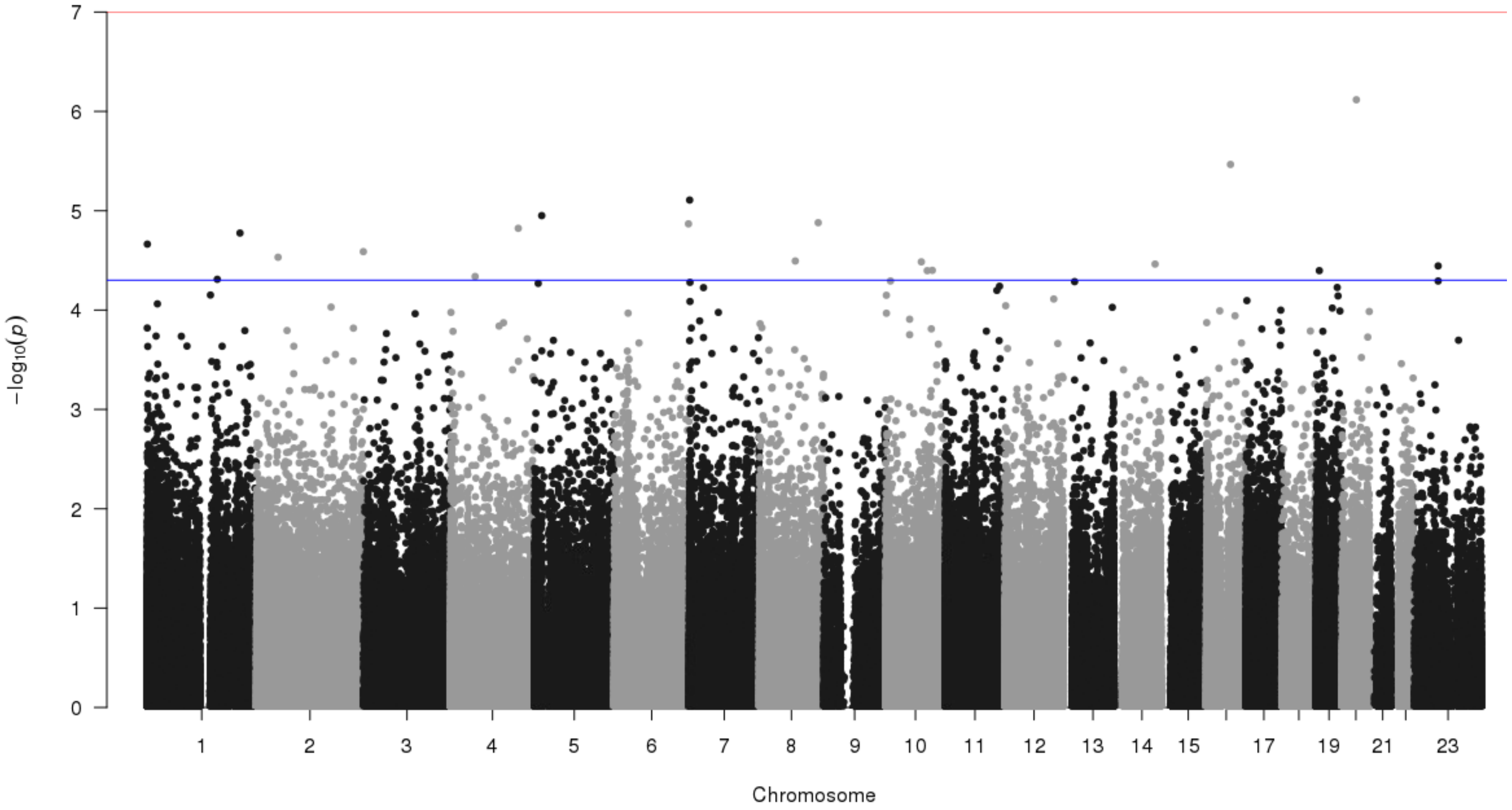

Figure S7. Boxplots showing case (red) and control (green) DNA methylation differences at the ten top-ranked differentially methylated positions (DMPs) associated with autism in the MINERvA cohort.

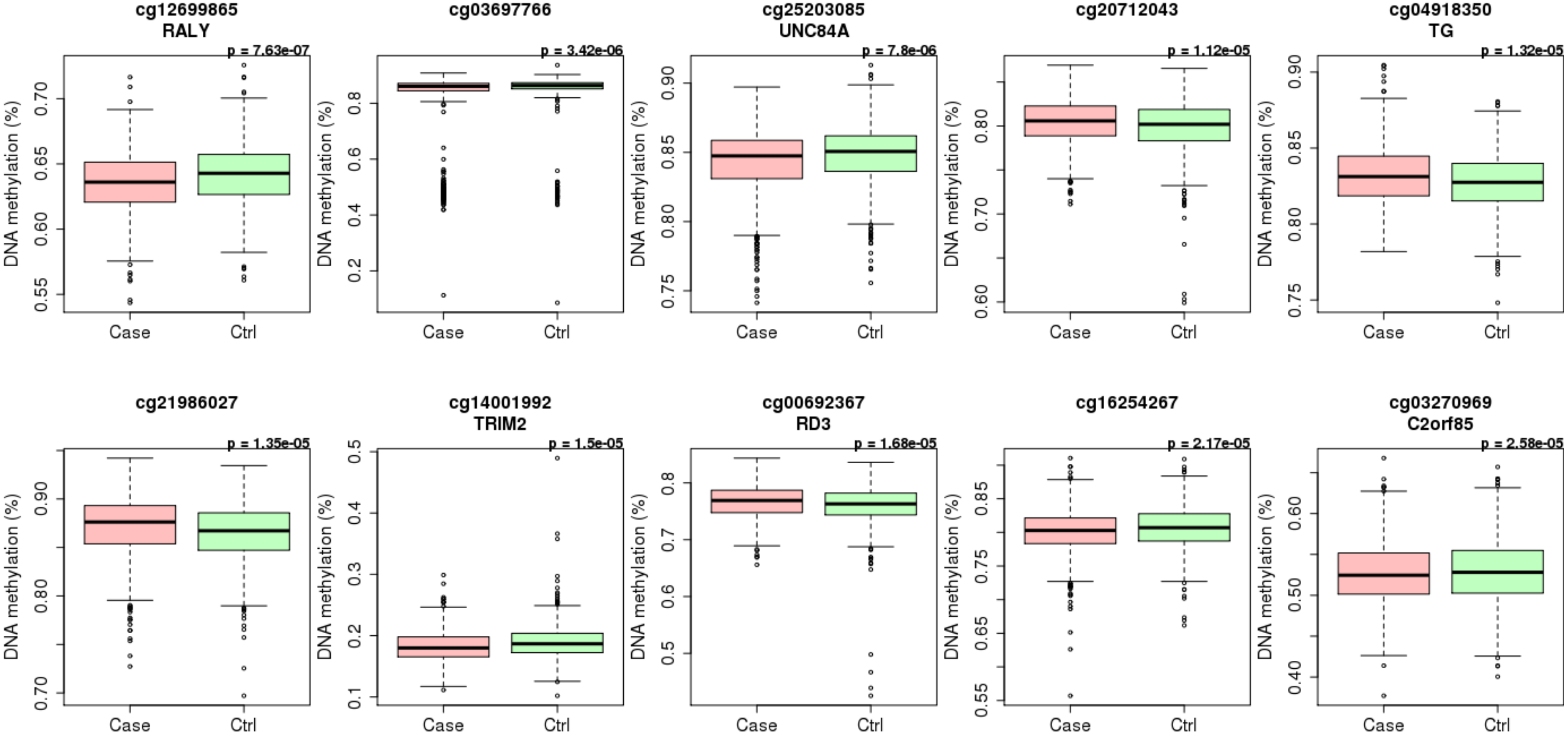

Figure S8. Scatterplots of autism-associated DNA methylation differences (% DNA methylation) at nominally-significant ($P < 5x10^{-5}$) DMPs identified in the MINERvA cohort (top row), SEED cohort (middle row) and Simons cohort (bottom row) against differences at the same DNA methylation sites in the other two other cohorts.

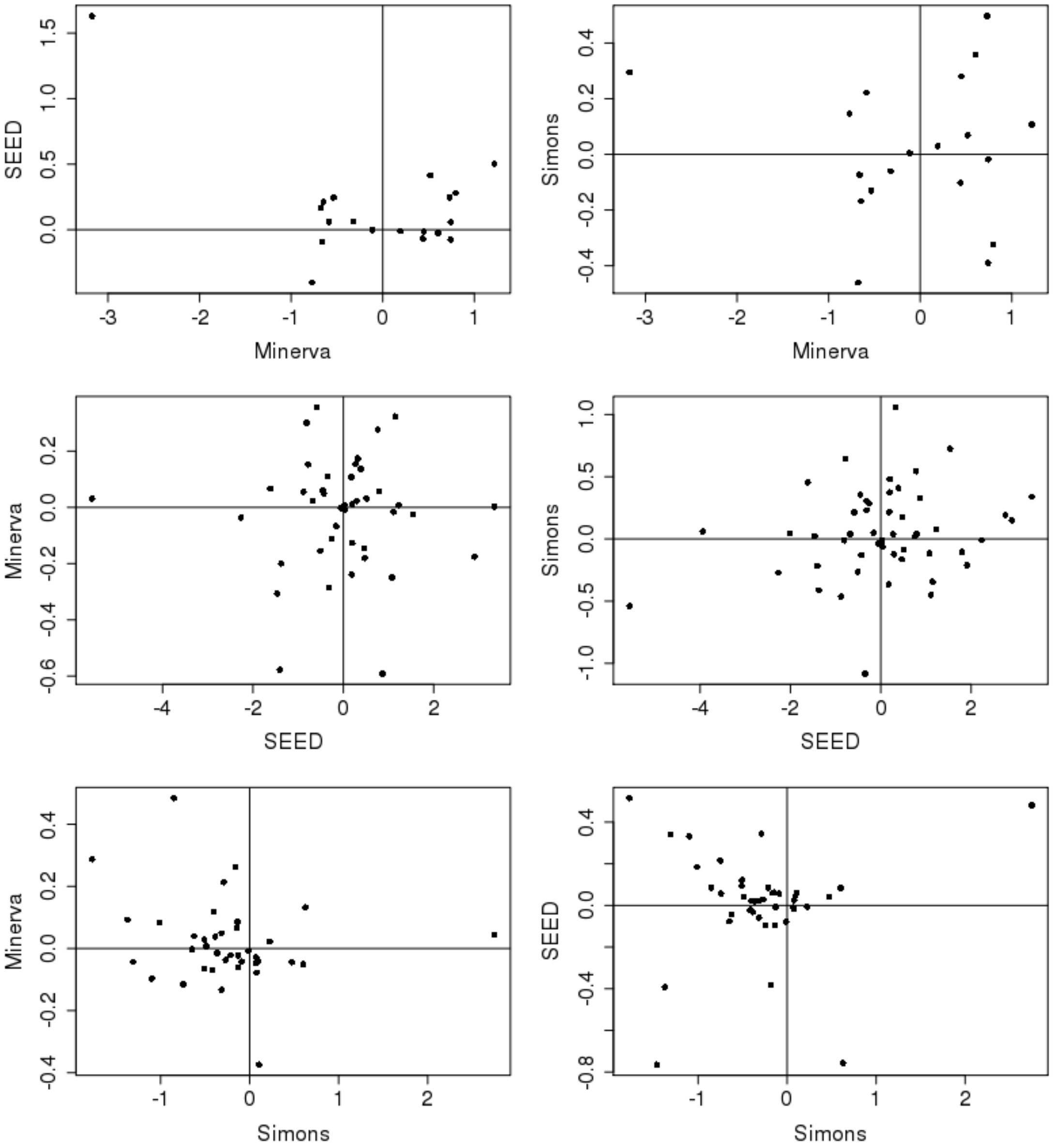

Figure S9. QQ-plot of P-values from the autism case-control EWAS meta-analysis. The meta-analysis included samples from the MINERvA, SEED and Simon's cohorts (total n = 2,917) (see **Materials and Methods**).

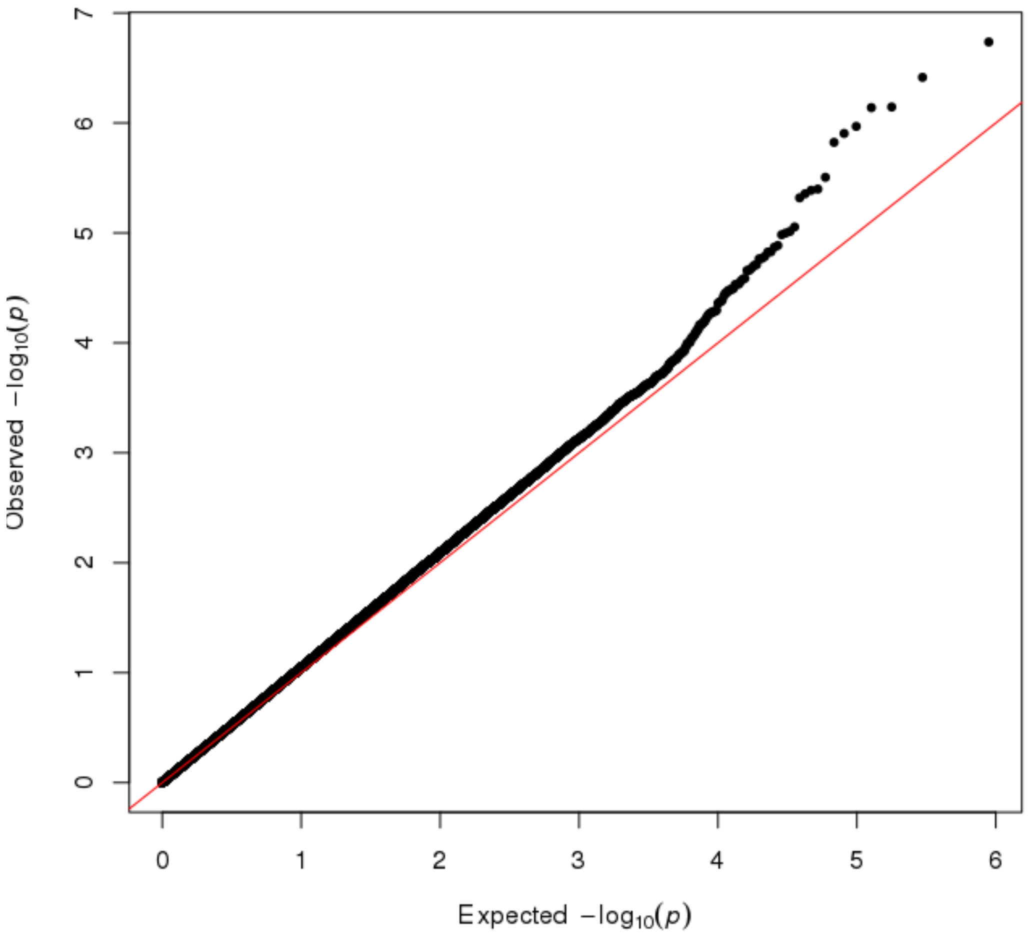

Figure S10. Forest plots for the four top-ranked autism-associated DMPs from the meta-analysis with consistent directions of effect. The effect is the mean difference in DNA methylation between autism cases and controls. The sizes of the boxes are proportional to the sample size of that cohort.

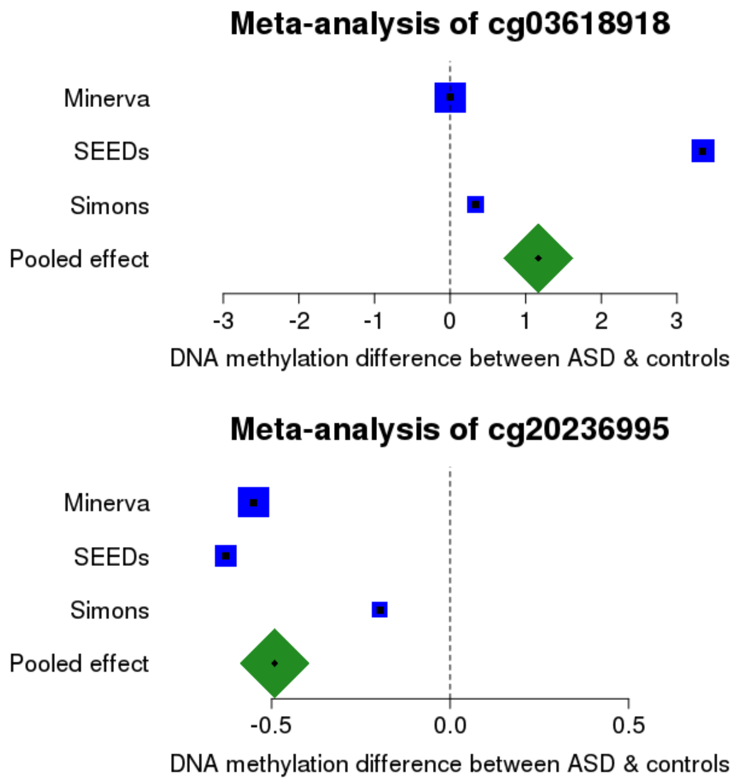

Figure S11. Density plots of autism polygenic risk score (PRS) estimates split by case (red) and control (green) status in the MINERvA cohort. Each box presents PRS calculated using a different P-value threshold (p_T) to define SNPs included in the calculation. S1 $p_T= 5x10^{-8}$; S2 $p_T= 1x10^{-6}$; S3 $p_T= 1x10^{-4}$; S4 $p_T=0.001$; S5 $p_T= 0.01$; S6 $p_T= 0.05$; S7 $p_T= 0.1$; S8 $p_T= 0.2$; S9 $p_T= 0.5$; S10 $p_T= 1$.

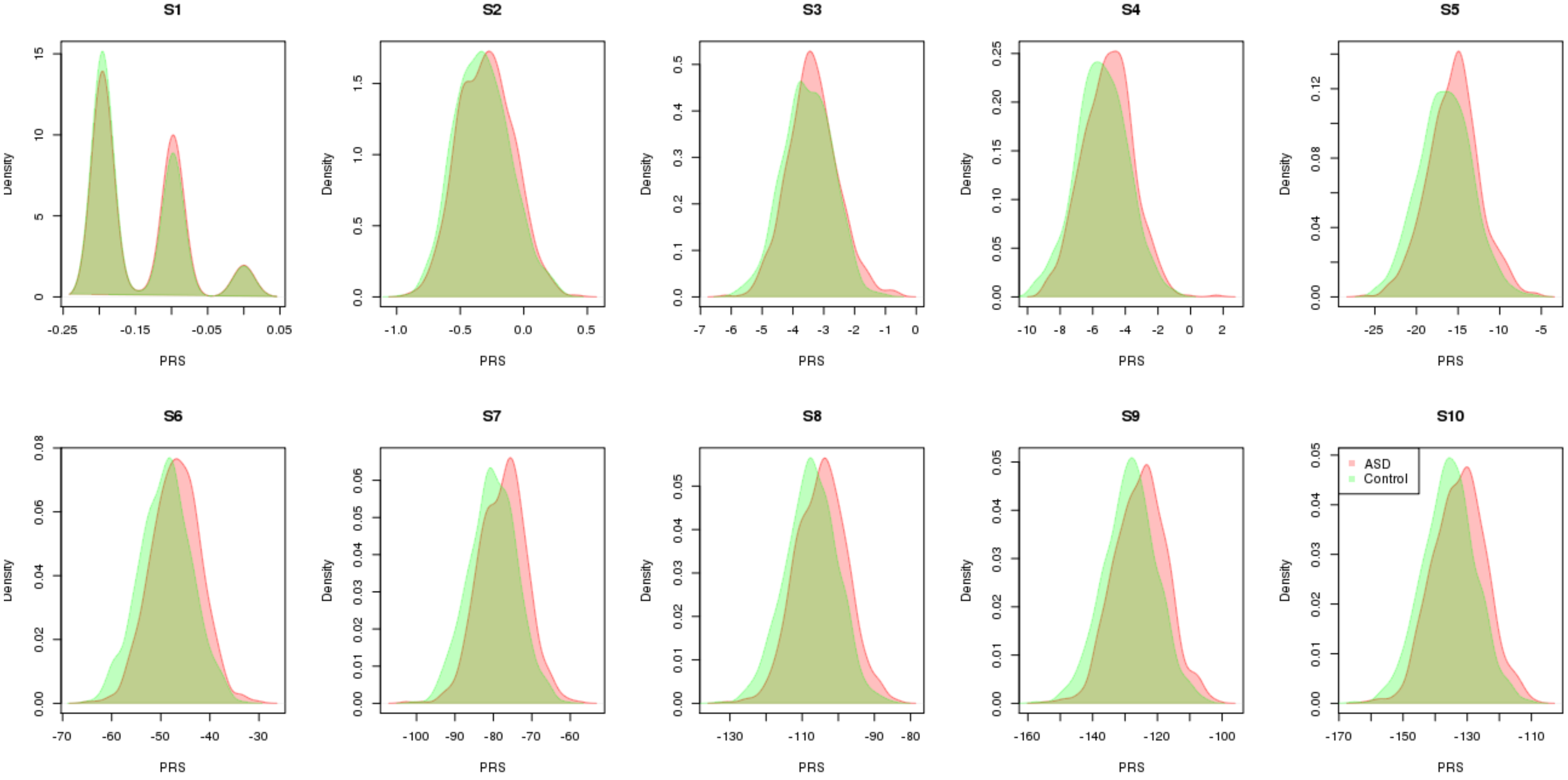

Figure S12. Correlation plot of autism polygenic risk scores (PRSs) calculated using different P-value thresholds (p_T) to define the group of SNPs included in each calculation. The boxes in the bottom triangle of the matrix contain scatterplots of PRS calculated at different significant thresholds with line of best fit. In the diagonal squares are histograms overlaid with a density plot of PRS at each threshold. The boxes in the top triangle contain the absolute correlation statistics with asterisks indicating the significance of the correlation statistic. S1 p_T= 5x10^{-8}; S2 p_T= 1x10^{-6}; S3 p_T= 1x10^{-4}; S4 p_T=0.001; S5 p_T= 0.01; S6 p_T= 0.05; S7 p_T= 0.1; S8 p_T= 0.2; S9 p_T= 0.5; S10 p_T= 1.

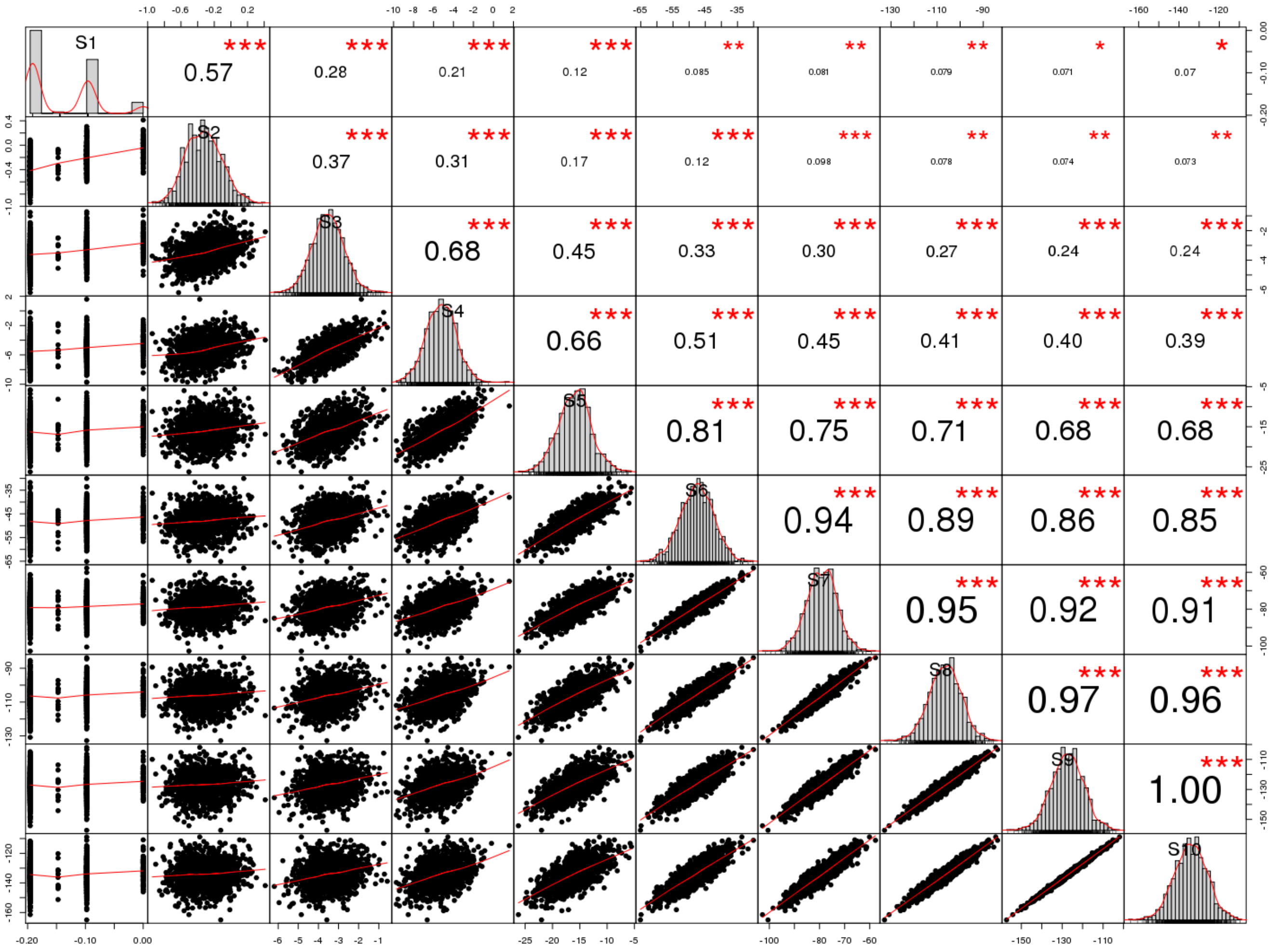

Figure S13. QQ plots of autism polygenic risk score (PRS) epigenome-wide association study (EWAS) analyses. Different panels present results using different P-value thresholds (p_T) to define SNPs included in the PRS calculation. S1 p_T= 5x10^{-8}; S2 p_T= 1x10^{-6}; S3 p_T= 1x10^{-4}; S4 p_T=0.001; S5 p_T= 0.01; S6 p_T= 0.05; S7 p_T= 0.1; S8 p_T= 0.2; S9 p_T= 0.5; S10 p_T= 1.

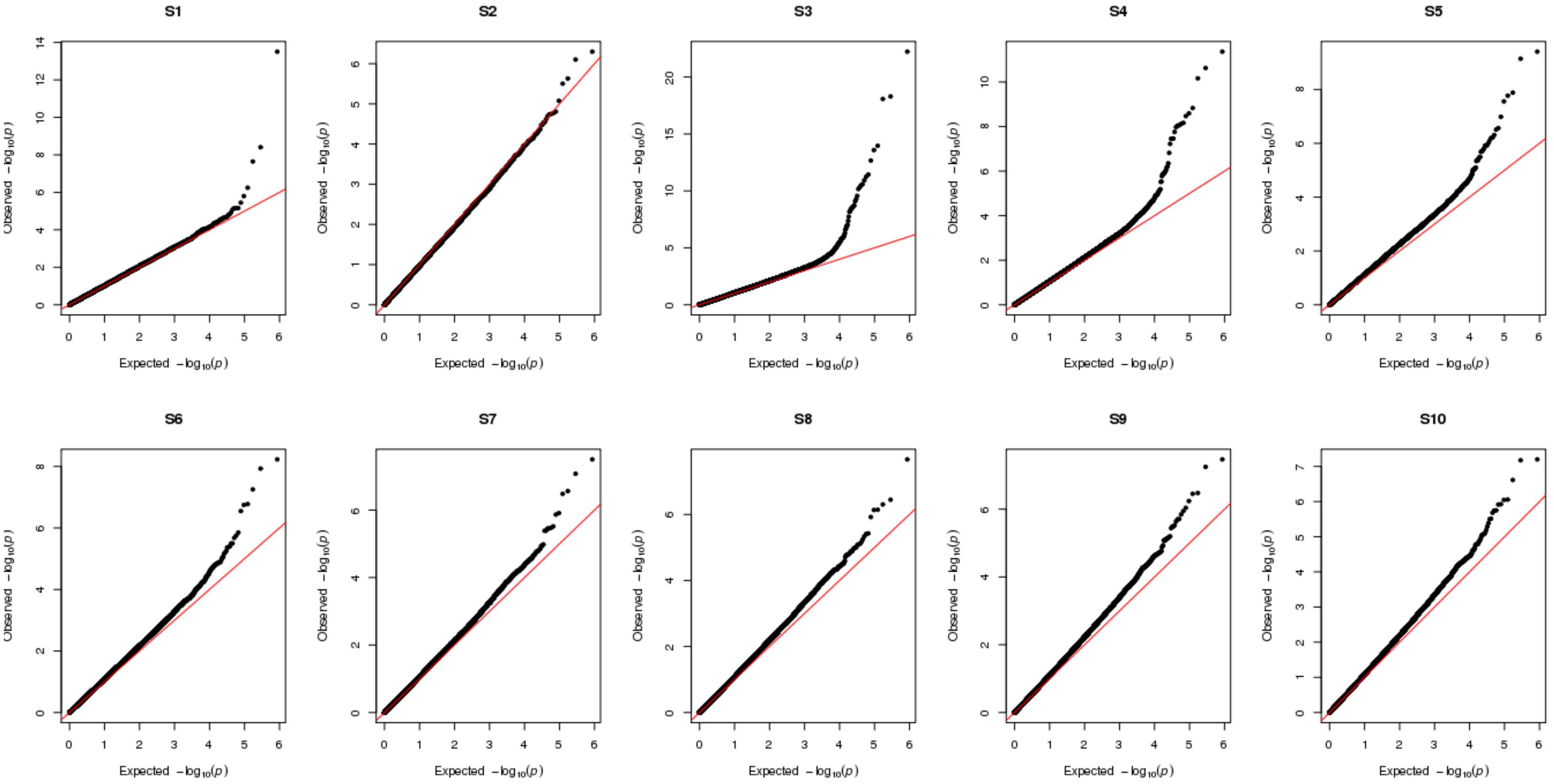

Figure S14. **Manhattan plots of P-values from the autism PRS EWAS calculated using different P-value thresholds (p_T).** The red horizontal line indicates experiment-wide significance ($P < 1x10^{-7}$). The blue horizontal line indicates a suggestive significance threshold ($P < 5x10^{-5}$). Results are shown for analyses using different P-value thresholds (p_T) to define SNPs included in the PRS calculation S1 p_T= $5x10^{-8}$; S2 p_T= $1x10^{-6}$; S3 p_T= $1x10^{-4}$; S4 p_T=0.001; S5 p_T= 0.01; S6 p_T= 0.05; S7 p_T= 0.1; S8 p_T= 0.2; S9 p_T= 0.5; S10 p_T= 1.

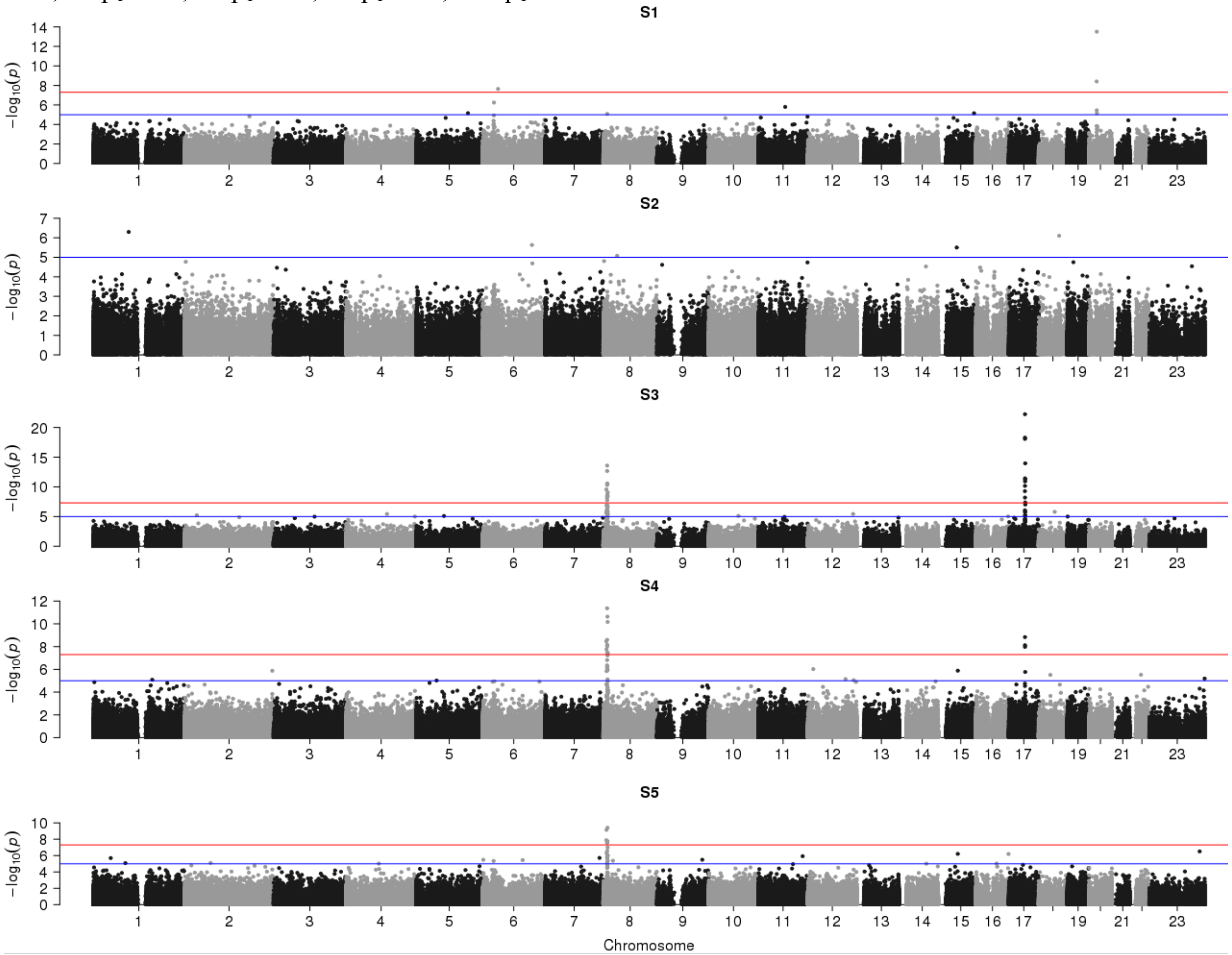

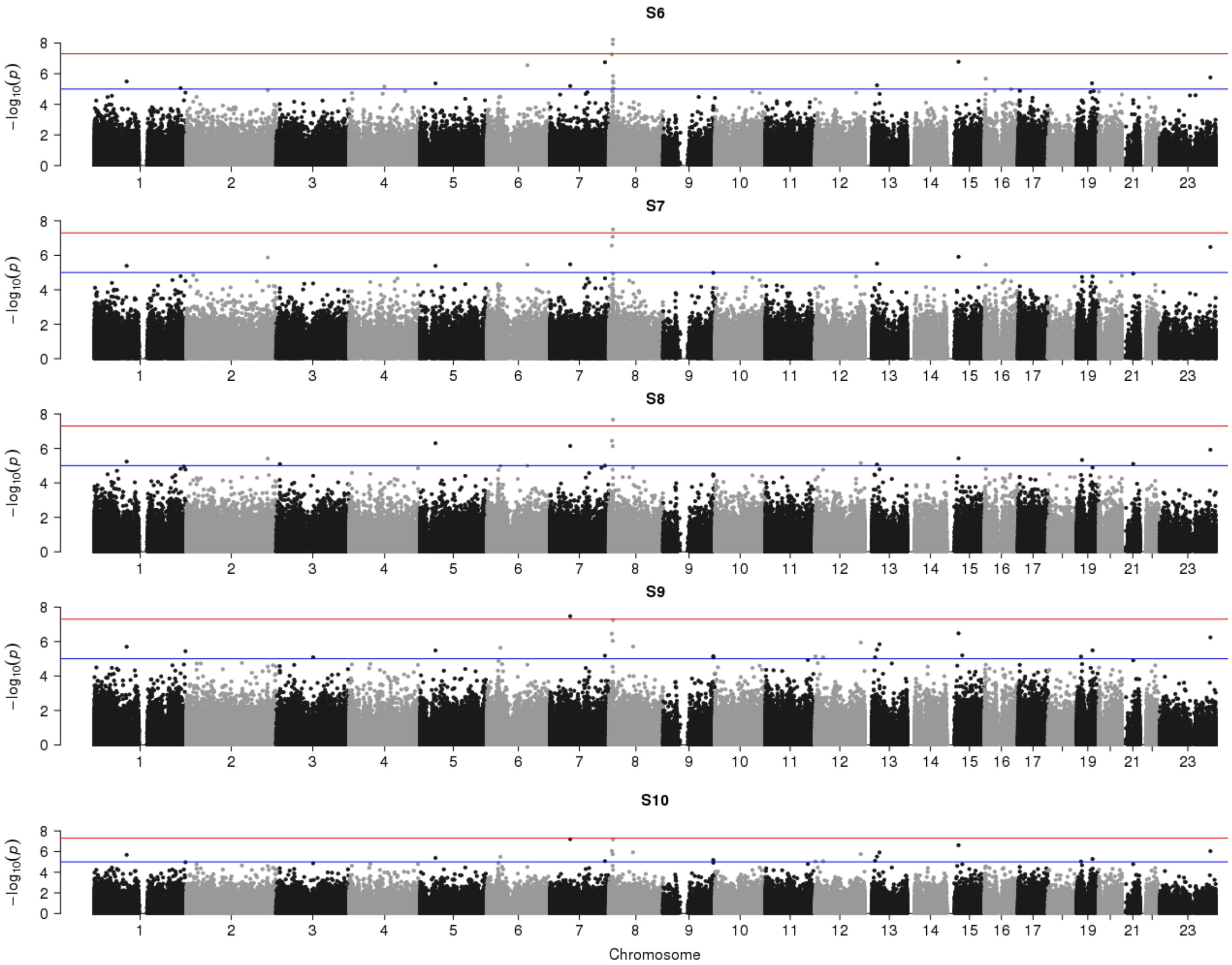
S6
S7
S8
S9
S10
−log10(p)
0
2
4
6
8
1
2
3
4
5
6
7
8
9
10
11
12
13
14
15
16
17
19
21
23
Chromosome

Figure S15. Correlation plot of –log10(P-values) from EWASs of the autism PRS calculated using different P-value thresholds (p_T). The boxes in the bottom triangle of the matrix contain scatterplots of PRSs calculated at different significant thresholds with line of best fit. In the diagonal squares are histograms overlaid with density plot of PRS at each threshold. The boxes in the top triangle contain the absolute correlation statistics with asterisks indicating the significance of the correlation statistic. S1 p_T= 5x10^{-8}; S2 p_T= 1x10^{-6}; S3 p_T= 1x10^{-4}; S4 p_T=0.001; S5 p_T= 0.01; S6 p_T= 0.05; S7 p_T= 0.1; S8 p_T= 0.2; S9 p_T= 0.5; S10 p_T= 1.

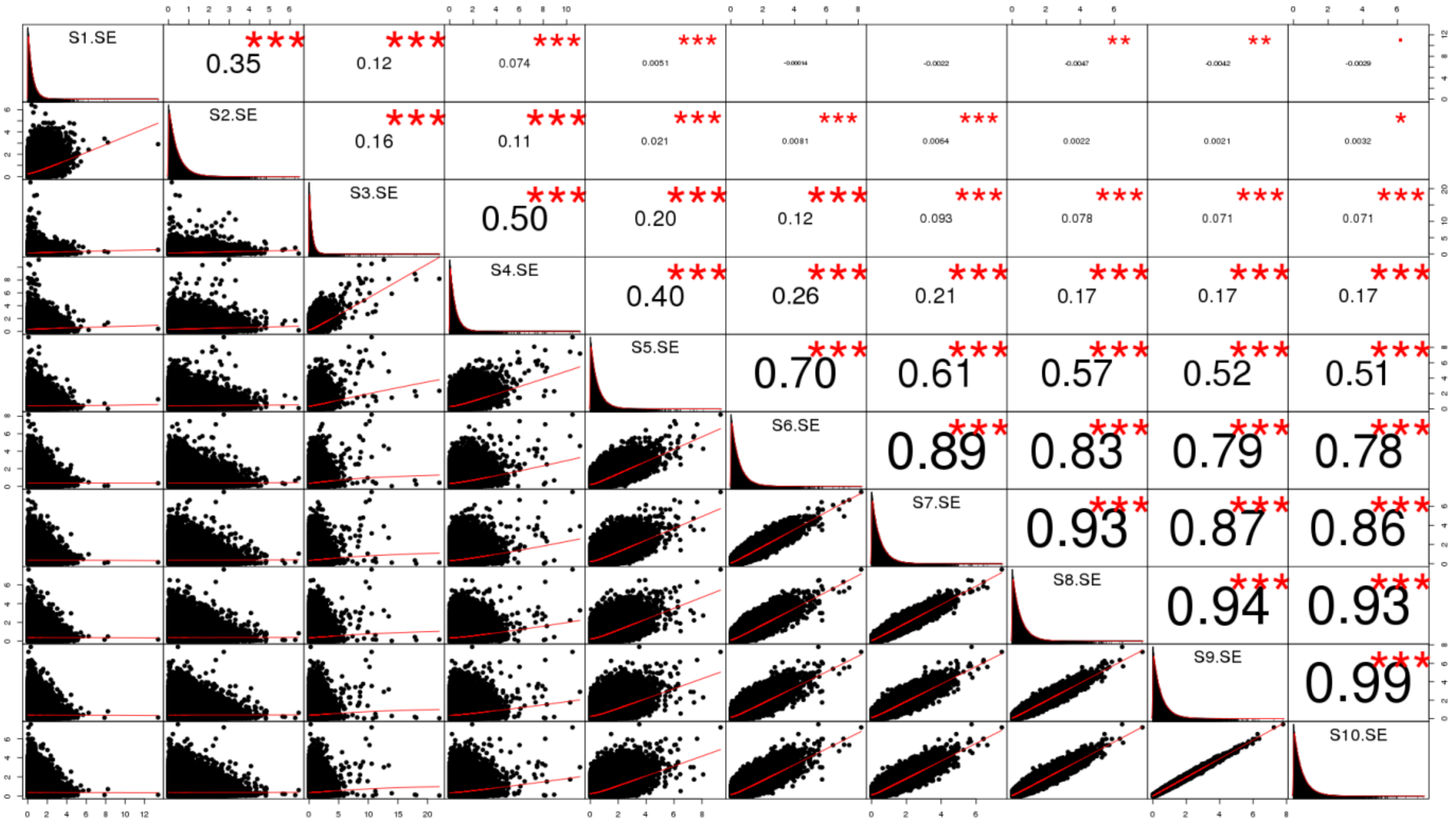

Figure S16. Scatterplots of autism PRS (calculated using the optimal threshold of pT < 0.1) against DNA methylation for the ten top-ranked DMPs identified in the EWAS of autism PRS. Points are colored to distinguish between autism cases (green) from controls (red).

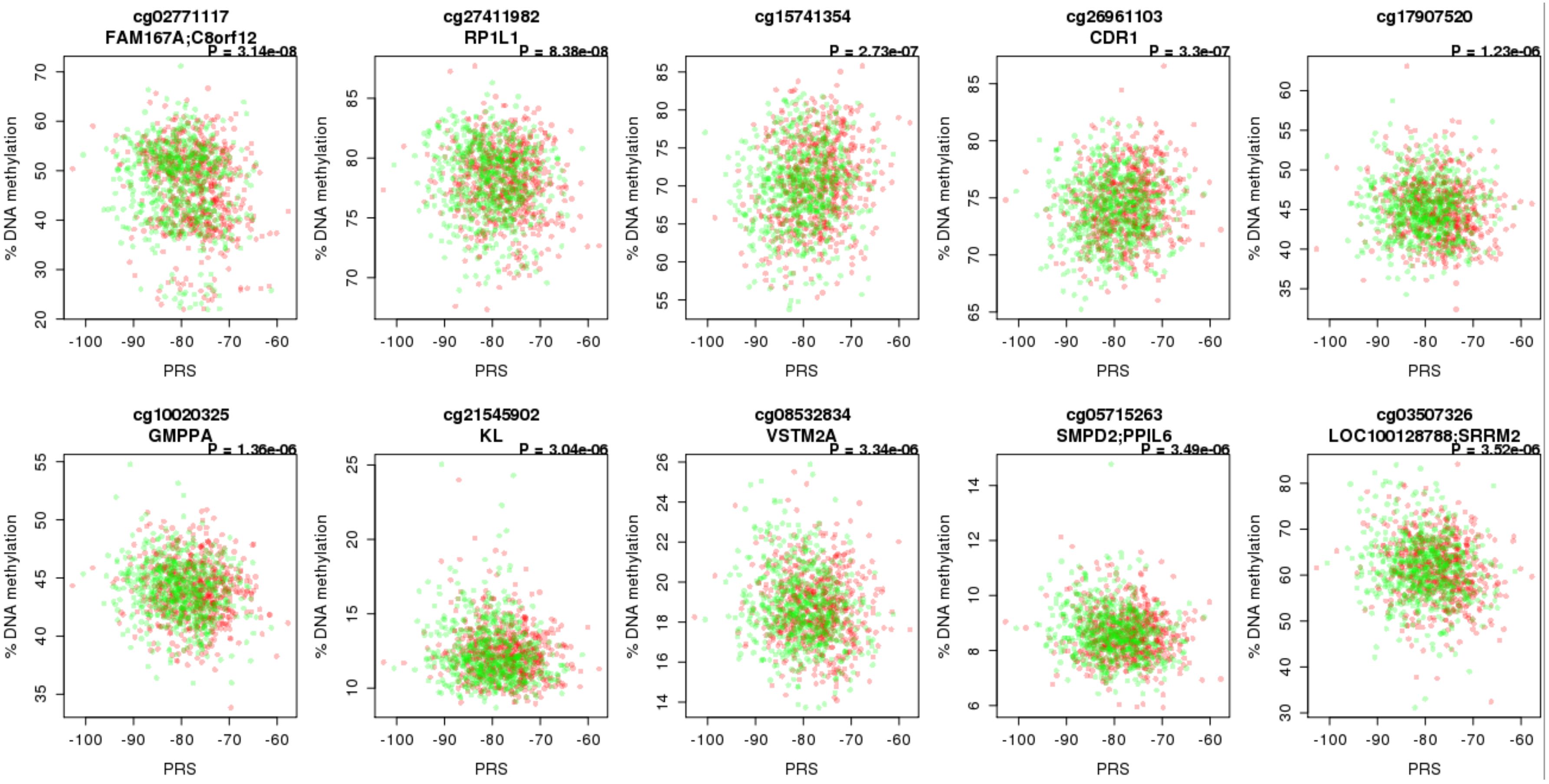

Figure S17. Manhattan plots of P-values from GWAS and EWAS of autism across a region on chromosome 8. A) Manhattan plot of GWAS of autism status. B) Manhattan plot of EWAS of ASD status. C) EWAS of autism polygenic risk score. Red horizontal lines indicate genome-wide significance for the GWAS ($P < 5x10^{-8}$) and experiment-wide significance for the EWAS ($P < 1x10^{-7}$). Genomic locations are based on hg19.

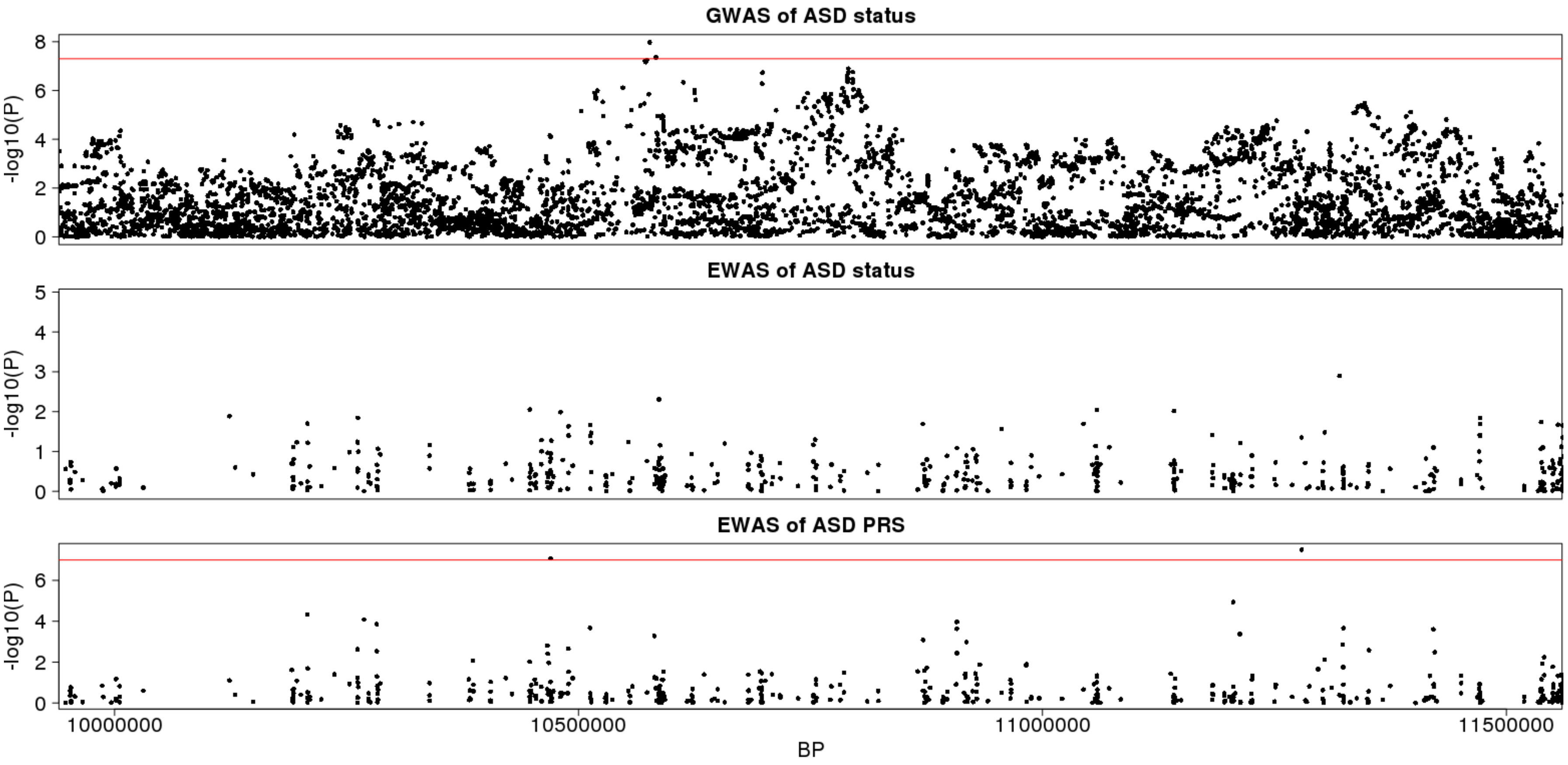

Figure S18. Regional plot of regulatory activity around the chromosome 8 region implicated in ASD by both GWAS and EWAS. Chromatin states as predicted by ChromHMM (15 state model) for blood and brain cell types and tissues centred around A) the index SNP in the chromosome 8 region from GWAS of ASD, and the two DNA methylation sites associated with ASD PRS: B) cg02771117 and C) cg27411982. Figure produced using UCSC Genome Browser and imputed chromHMM tracks from the Roadmpa Epigenomics Project. Genomic locations are based on hg19.

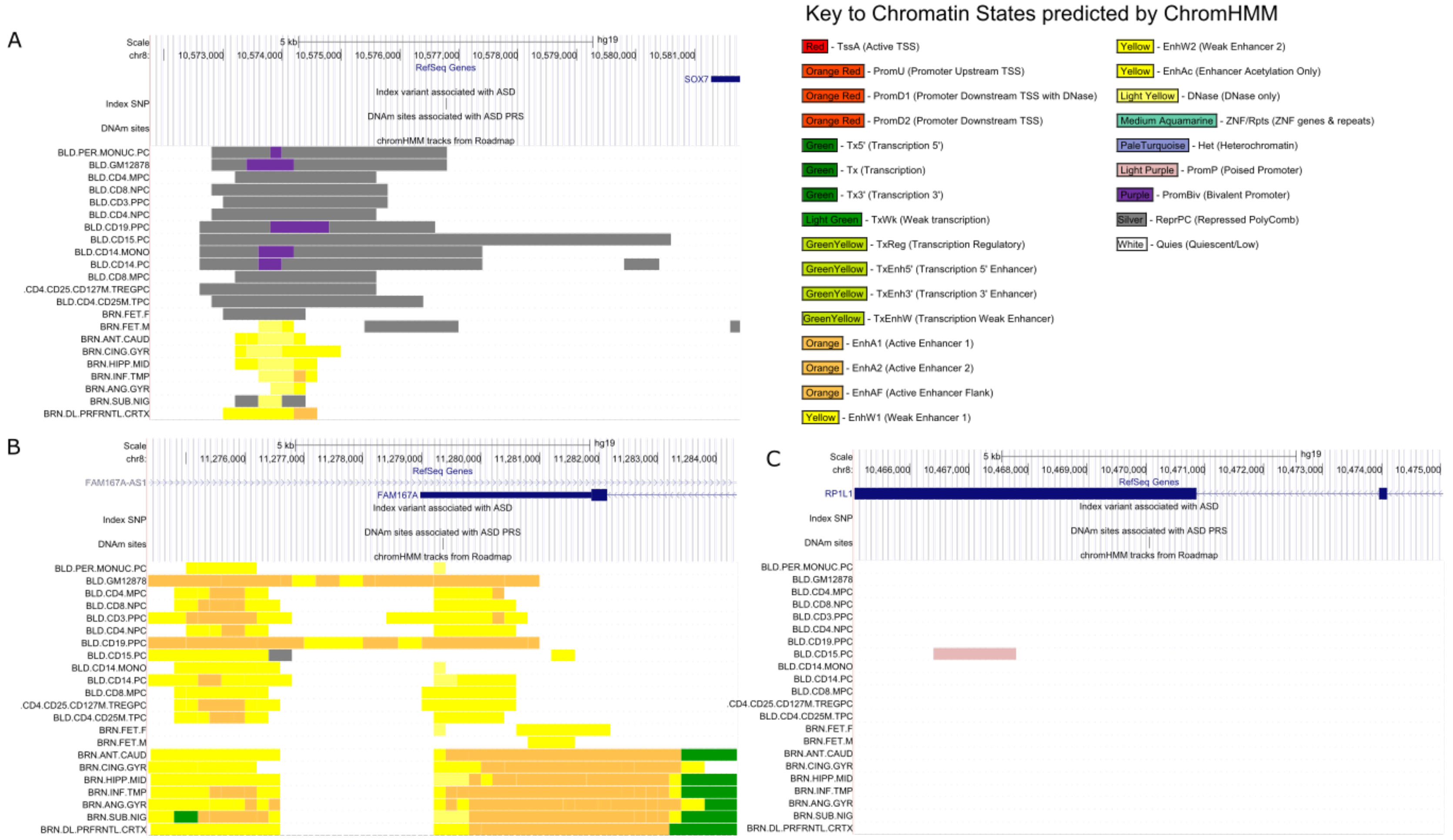

Roadmap Epigenomics Consortium, Kundaje, A., Meuleman, W., Ernst, J., Bilenky, M., Yen, A., Heravi-Moussavi, A., Kheradpour, P., Zhang, Z., Wang, J., et al. (2015). Integrative analysis of 111 reference human epigenomes. Nature 518, 317-330.

Figure S19. Boxplots of DNA methylation at cg02771117 split by genotype at genetic variants in the associated chromosome 8 region included in the ASD PRS. Each boxplot presents an association between a genetic variant (x-axis) and DNA methylation at cg02771117 (y-axis), the p value reported is taken from a linear regression analysis between these variables.

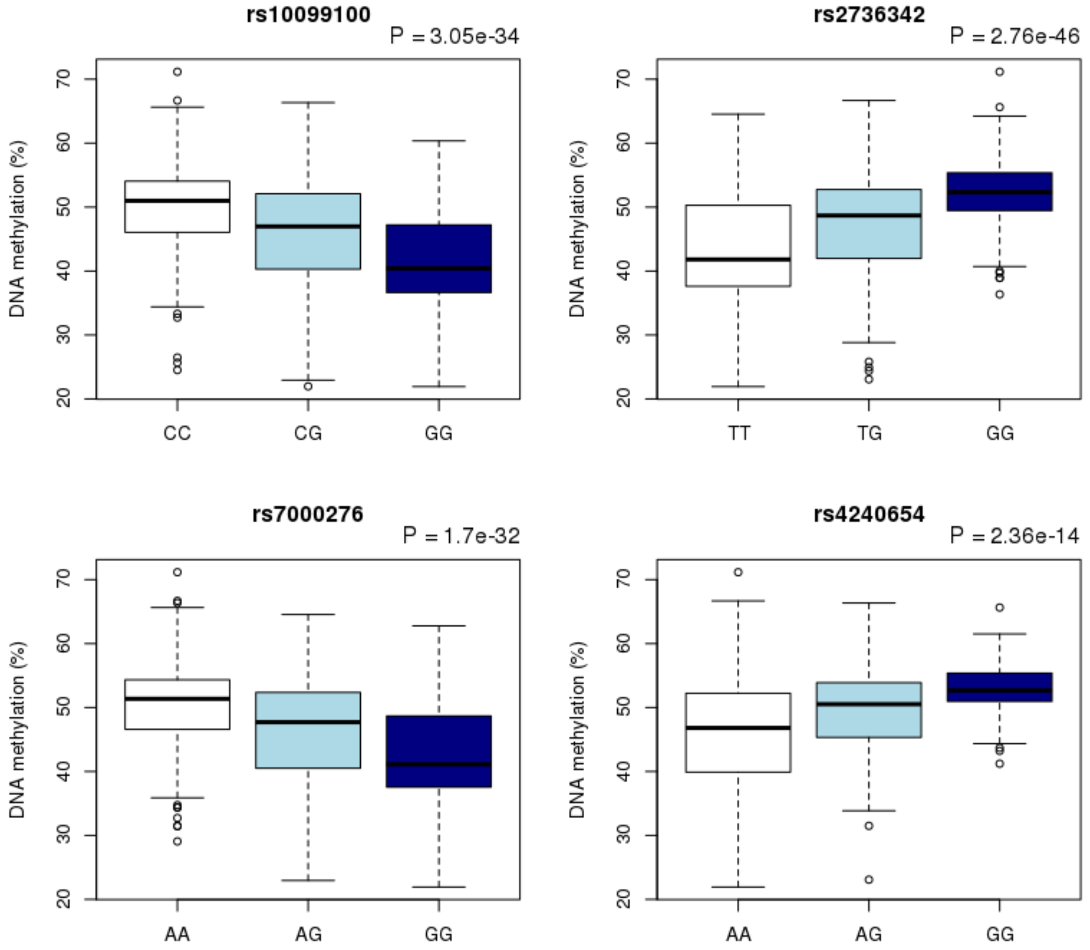

Figure S20. Boxplots of DNA methylation at cg27411982 split by genotype at genetic variants in the associated chromosome 8 region included in the ASD PRS. Each boxplot presents an association between a genetic variant (x-axis) and DNA methylation at cg27411982 (y-axis), the p value reported is taken from a linear regression analysis between these variables.

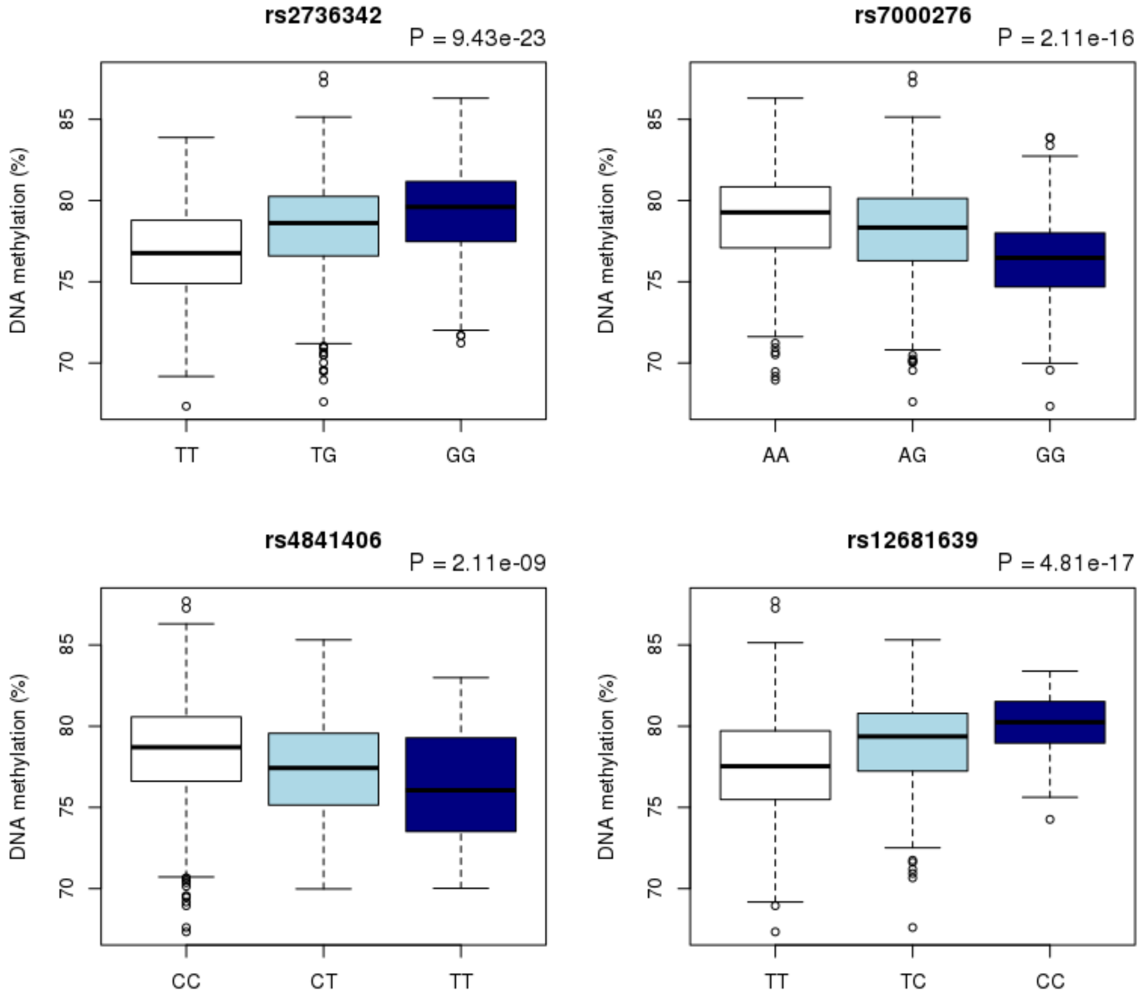

Figure S21. Scatterplots of –log10 P-values from the EWAS of ASD PRS comparing analysis performed in all individuals (x-axis) against analysis performed separately for cases and controls and then combined with a meta-analysis (y-axis).

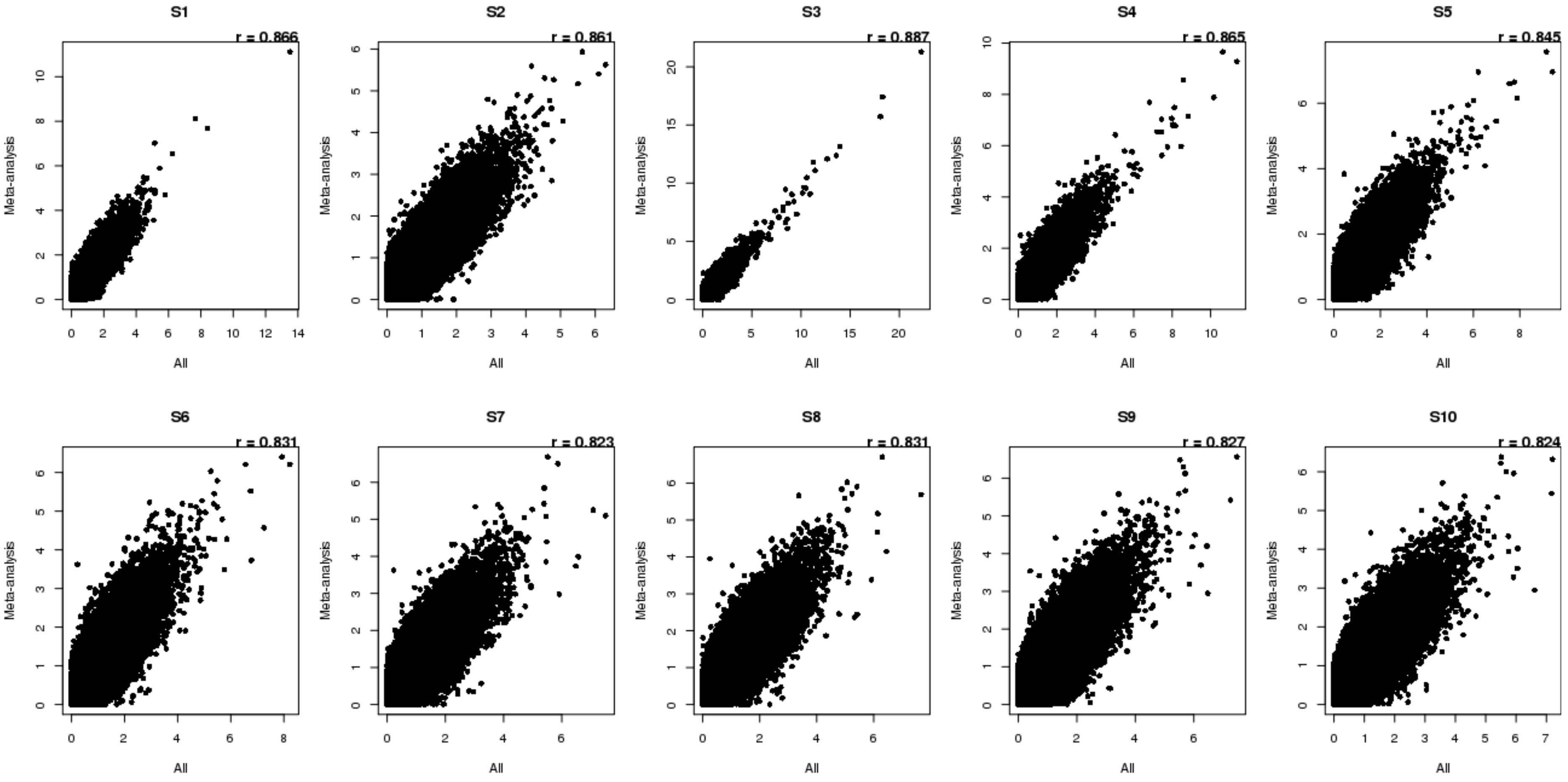

Figure S22: mQTL mapping can localize putative causal loci associated with disease. Presented here is are two genomic regions (chr20:14836243-14926587 and chr20:21233198-21494184) identified in a recent GWAS analysis of ASD. At the top of the figure is a schematic detailing which genes are located in these regions which are identified by their Entrez ID. All genetic variants identified in the largest GWAS of ASD to date ($P < 1x10^{-4}$) (Grove et al, submitted) are represented by vertical solid lines where the colour reflects the strength of the association ranging from gray (less significant P-values) to black (more significant P-values); a red vertical line indicates the most significant genetic variant in this region. All DNA methylation sites tested for mQTL in the Minerva dataset are indicated by red vertical lines and genetic variants by blue vertical lines. Significant mQTL ($P < 1x10^{-13}$) are indicated by black diagonal lines between the respective genetic variant and DNA methylation site. Genomic locations are based on hg19.

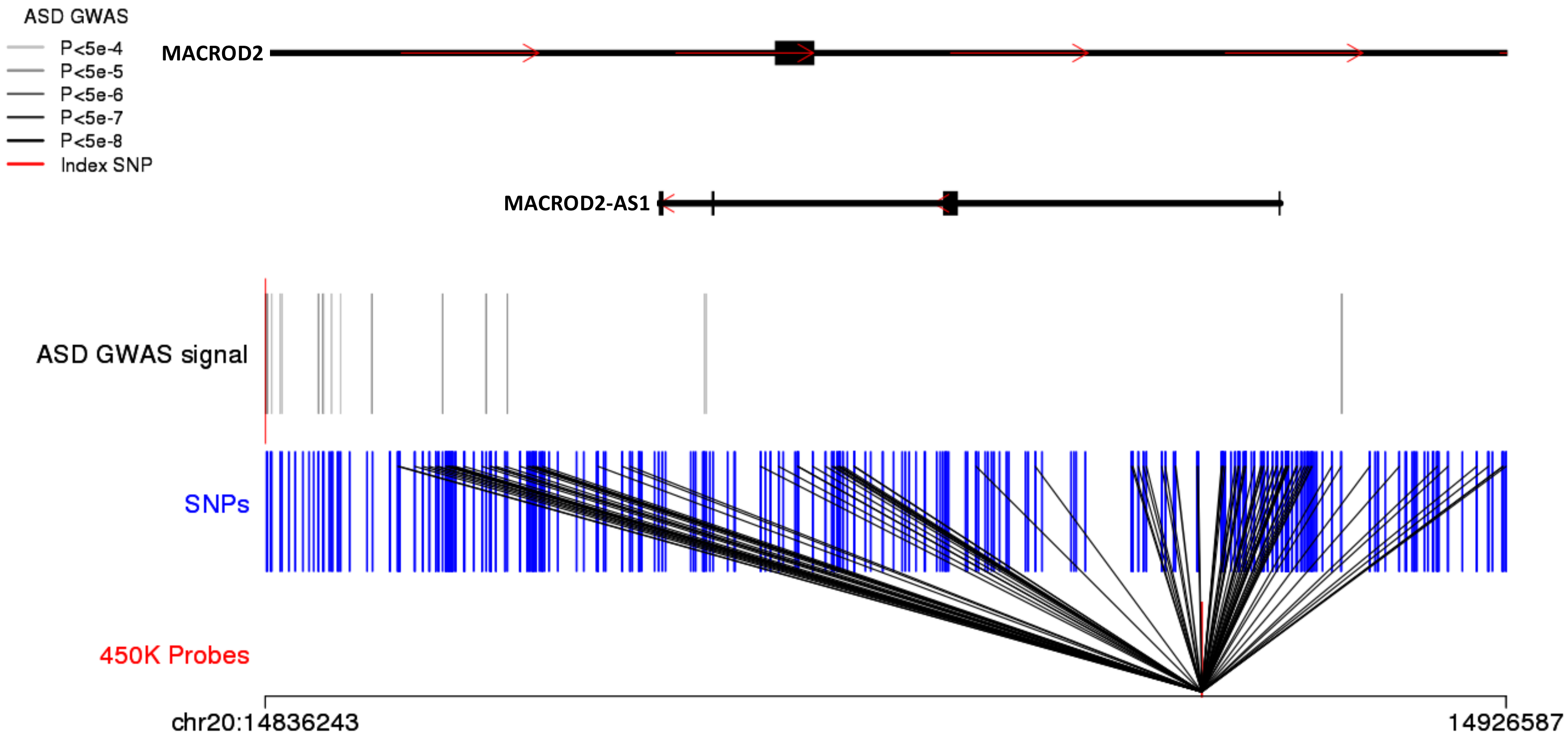

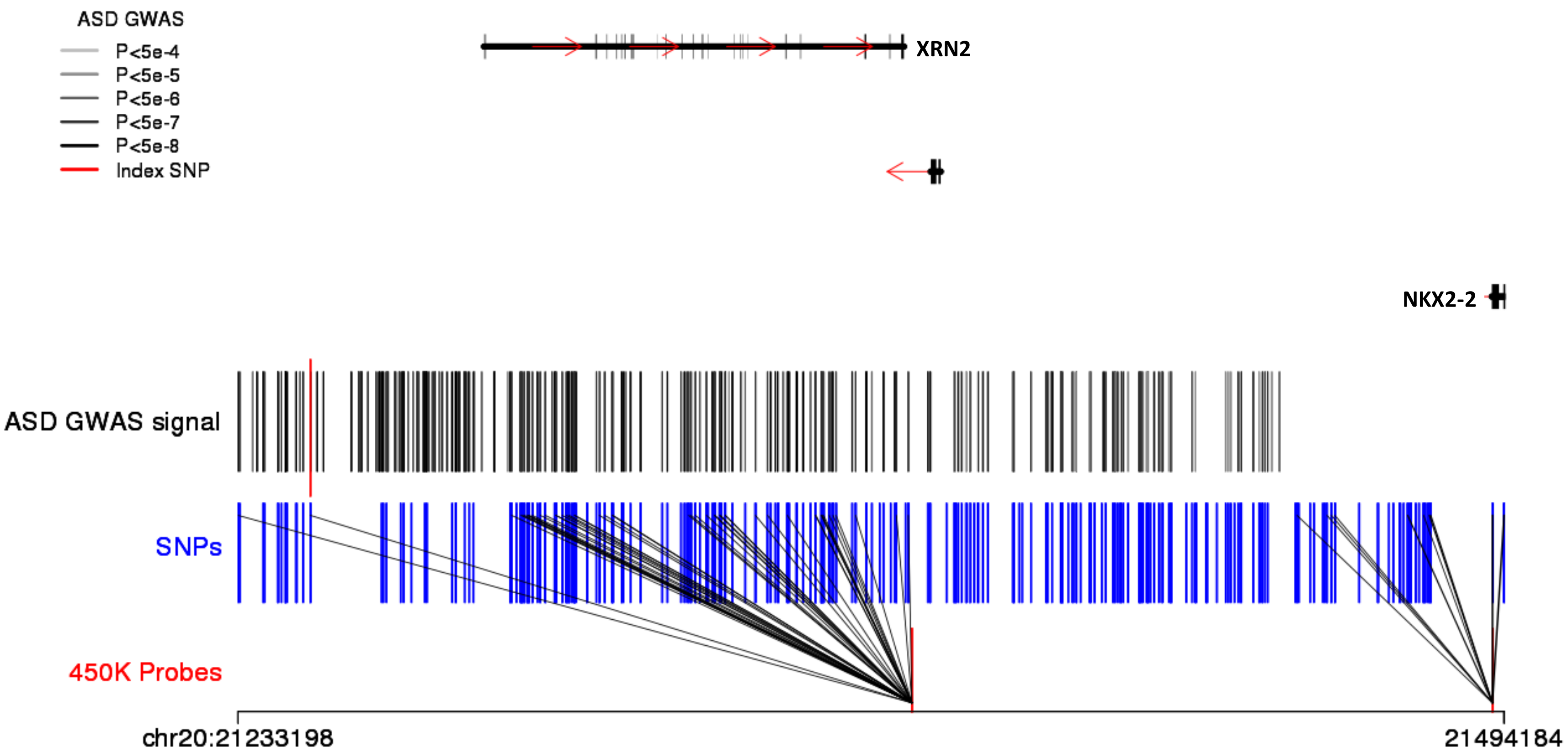

ASD GWAS
P<5e-4
P<5e-5
P<5e-6
P<5e-7
P<5e-8
Index SNP
XRN2
NKX2-2
ASD GWAS signal
SNPs
450K Probes
chr20:21233198
21494184

Figure S23: Evidence for co-localisation of ASD and DNA methylation. Region on chromosome 20 where four CpGs sites had genetic signals consistent with the same causal variant being associated with both ASD and DNA methylation. Manhattan plots of the GWAS signal for A) ASD, B) cg26861732, C) cg04219410, D) cg00239420, and E) cg01798261 in this region (chr20:20906687-21578495). In B-E the red vertical line indicates the location of the CpG site. Genomic locations are based on hg19.

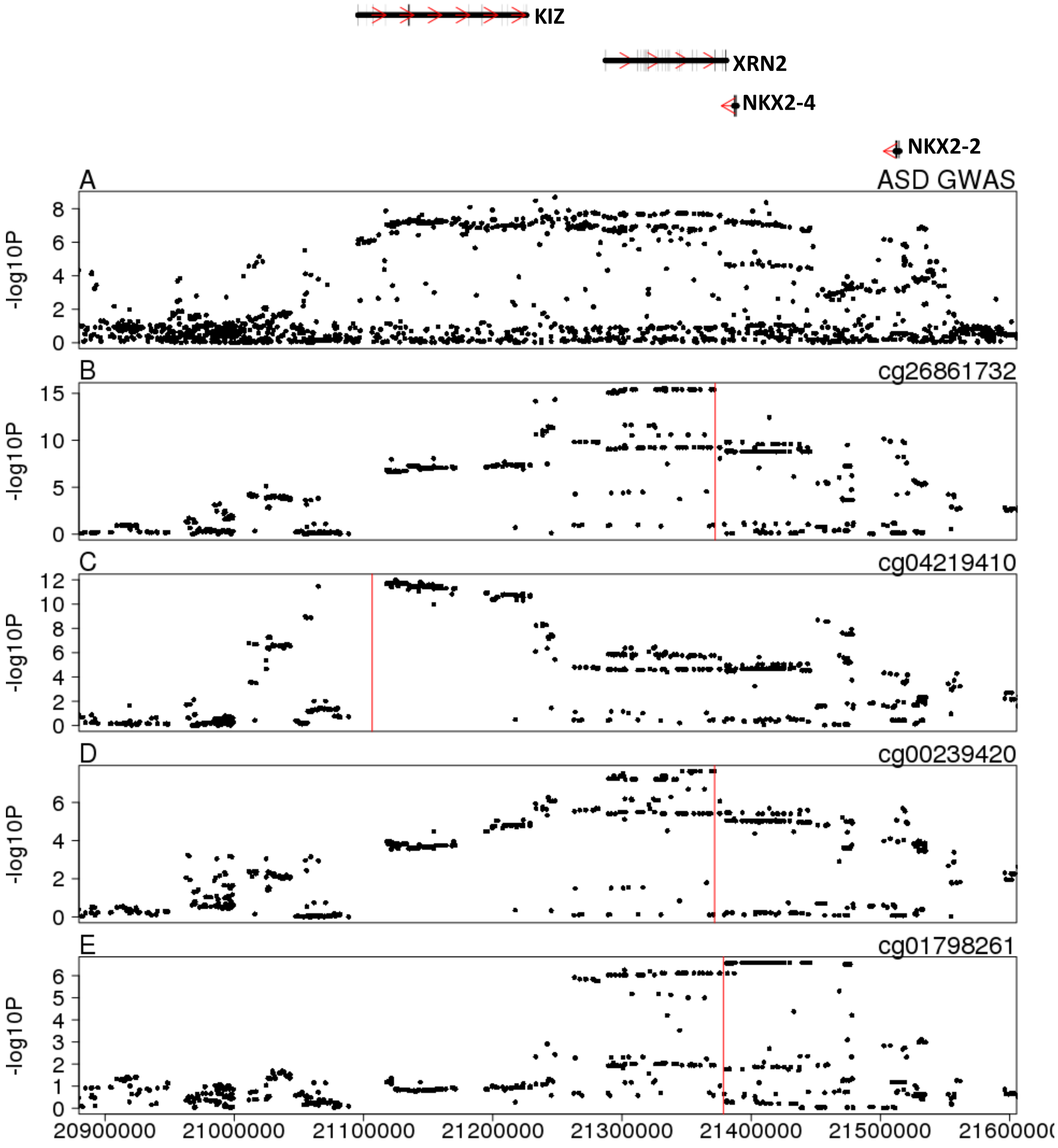

Supplement: Supplementary file 1 — Supplementary Figures S1–S23. (PDF 8163 kb) [file 13073_2018_527_MOESM1_ESM.pdf]
