# Supplementary material for: Elevated polygenic burden for autism is associated with differential DNA methylation at birth
Source: Genome Med. 2018 Mar 28;10:19. doi: 10.1186/s13073-018-0527-4 (PMC5872584; doi:10.1186/s13073-018-0527-4)
Supplement: Supplementary file 2 — Supplementary Tables S1–S7 and S9–S10. (PDF 339 kb) [file 13073_2018_527_MOESM2_ESM.pdf]

Table S1. Diagnostic codes from the World Health Organization (WHO) International Classification of Diseases version 10 (ICD-10) used to select and exclude samples for methylomic profiling as either ASD cases or controls.

**Autism spectrum disorder**	
**F84.0**	Autistic disorder
**F84.1**	Atypical autism
**F84.5**	Asperger syndrome
**F84.8**	Other pervasive developemental disorders
**F84.9**	Pervasive developmental disorder, unspecified
**F00-F99**	**Any psychiatric disorder**
**Genetic conditions**	
**D82.1**	DiGeorgi syndrome
**G71.0**	muscular dystrophy
**Q85.0**	neurofibromatosis
**Q85.1**	tuberous sclerosis
**Q87.1**	De Lange/Prader-Willi/Smith-Lemli-Opitz
**Q87.8**	Zellweger syndrome
**Q90**	Down syndrome
**Q93.5**	Angelman syndrome
**Q93.8**	Williams syndrome
**Q98.0-98.4**	Klinefelter syndrome
**Q99.2**	Fragile X syndrome

Table S2. Twenty ASD-associated DMPs were identified at a "discovery" threshold of $P < 5x10^{-5}$. Genomic locations are based on hg19.

Probe ID	ASD EWAS in Minerva			Chr	Pos	Gene Annotation	
	P-value	Mean Diff	SE			UCSC RefGene Name	UCSC RefGene Group
cg12699865	7.63E-07	-0.65%	0.13%	chr20	32583031	RALY;RALY	5'UTR;5'UTR
cg03697766	3.42E-06	-3.17%	0.68%	chr16	54848022		
cg25203085	7.80E-06	-0.59%	0.13%	chr7	887678	UNC84A;UNC84A	Body;Body
cg20712043	1.12E-05	0.60%	0.14%	chr5	16392700		
cg04918350	1.32E-05	0.44%	0.10%	chr8	134124199	TG	Body
cg21986027	1.35E-05	0.74%	0.17%	chr6	169238138		
cg14001992	1.50E-05	-0.68%	0.16%	chr4	154073813	TRIM2	TSS1500
cg00692367	1.68E-05	0.80%	0.18%	chr1	211665511	RD3;RD3;RD3;RD3	1stExon;5'UTR;1stExon;5'UTR
cg16254267	2.17E-05	-0.77%	0.18%	chr1	1073529		
cg03270969	2.58E-05	-0.66%	0.16%	chr2	242813189	C2orf85	Body
cg06995408	2.94E-05	0.73%	0.17%	chr2	48977089	LHCGR	Body
cg09973676	3.20E-05	1.22%	0.29%	chr8	82006417	PAG1	5'UTR
cg25485956	3.27E-05	-0.11%	0.03%	chr10	81146099	ZCCHC24	3'UTR
cg23256480	3.44E-05	0.19%	0.05%	chr14	93252030		
cg22829182	3.59E-	0.75%	0.18%	chrX	56258808	KLF8;KLF8	TSS200;TSS200

	05						
cg02803139	3.98E-05	-0.32%	0.08%	chr10	106113391	CCDC147	TSS200
cg03260991	4.01E-05	-0.54%	0.13%	chr19	7539710	LOC100128573	TSS1500
cg04089434	4.01E-05	0.52%	0.13%	chr10	94516971		
cg02723107	4.59E-05	0.45%	0.11%	chr4	55987799	KDR	Body
cg20064848	4.90E-05	0.74%	0.18%	chr1	160037877	KCNJ10	5'UTR

Table S3. Seven DMPs characterized by an interaction between autism status and sex were identified at our "discovery" threshold of $P < 5x10^{-5}$. Genomic locations are based on hg19.

Probe ID	ASD EWAS in females			ASD EWAS in males			ASD EWAS with sex interaction									Chr	Pos
							ASD main effect			Sex main effect			Interaction				
	P-value	Mean Diff	SE	P-value	Mean Diff	SE	P-value	Mean Diff	SE	P-value	Mean Diff	SE	P-value	Mean Diff	SE		
cg00181669	0.00117	-0.59%	0.18%	0.0071	0.50%	0.18%	0.0014	-0.58%	0.18%	0.0016	-0.61%	0.19%	2.76E-05	1.09%	0.26%	chr3	44000978
cg01987333	0.03174	-0.41%	0.19%	0.0003	0.65%	0.18%	0.0249	-0.42%	0.19%	0.0798	-0.34%	0.19%	4.38E-05	1.07%	0.26%	chr2	242750561
cg08073882	0.04848	-0.09%	0.05%	0.0001	0.19%	0.05%	0.0486	-0.10%	0.05%	0.7944	0.01%	0.05%	3.42E-05	0.29%	0.07%	chr4	6202381
cg10911747	0.02249	0.30%	0.13%	0.0013	-0.47%	0.14%	0.0194	0.32%	0.13%	0.0906	0.24%	0.14%	3.91E-05	-0.79%	0.19%	chr4	15171497
cg13812927	0.00682	0.44%	0.16%	0.0014	-0.62%	0.19%	0.0099	0.46%	0.18%	0.0020	0.58%	0.19%	1.89E-05	-1.08%	0.25%	chr9	89517863
cg22518373	0.00080	0.58%	0.17%	0.0053	-0.46%	0.17%	0.0006	0.57%	0.17%	0.0088	0.46%	0.17%	3.37E-05	-0.98%	0.24%	chr17	77076358
cg23924222	0.02045	0.38%	0.16%	0.0026	-0.49%	0.16%	0.0123	0.40%	0.16%	0.0001	0.65%	0.17%	4.02E-05	-0.94%	0.23%	chr2	24658294

Table S4. Overview of samples included in our ASD DNA methylation meta-analysis. In total, we had data from a combined sample size of 1,487 ASD cases and 1,430 controls.

Variable	Measure	Minerva	SEED	Simons	Total
Sample number	Number with Illumina 450K data	1263	968	686	2917
Diagnosis	(% cases)	49.8	46.8	50	48.9

Table S5. All DMPs identified at a discovery significance threshold (P < 5e-5) in the MINERvA, SEED or Simons cohorts, and corresponding effects in the other cohorts. Genomic locations are based on hg19.

Probe ID	Minerva			SEED			Simon			Chr	Pos
	Mean Diff	SE	P-value	Mean Diff	SE	P-value	Mean Diff	SE	P-value		
cg00371627	0.02%	0.19%	0.90758	-0.01%	1.35%	0.511947	0.22%	1.86%	3.25E-06	chr7	77167295
cg00692367	0.80%	0.18%	1.68E-05	0.28%	1.98%	0.38582	-0.32%	1.70%	0.023078	chr1	211665511
cg00791854	0.11%	0.16%	0.512924	0.17%	0.97%	2.17E-06	-0.37%	1.31%	0.432839	chr1	154392070
cg01611626	-0.18%	0.21%	0.389549	0.48%	1.15%	4.24E-05	0.17%	1.48%	0.090573	chr16	2565338
cg01798266	0.05%	0.13%	0.713973	-0.43%	1.17%	2.39E-05	-0.13%	1.47%	0.012983	chr1	153320414
cg01951617	-0.25%	0.21%	0.224194	1.08%	1.32%	2.87E-05	-0.12%	1.48%	0.886709	chr2	121411889
cg02024846	0.09%	0.25%	0.712291	-0.39%	1.65%	0.819335	-1.37%	1.96%	4.95E-05	chr9	99382466
cg02556928	0.30%	0.23%	0.200525	-0.81%	2.01%	2.32E-05	-0.01%	2.01%	0.705955	chr22	37309980
cg02606018	NA	NA	NA	1.80%	0.87%	7.55E-07	-0.10%	0.87%	0.362562	chr12	10658281
cg02680487	0.13%	0.23%	0.566587	-0.76%	1.16%	0.547229	0.63%	1.00%	1.37E-05	chr6	30851529
cg02723107	0.45%	0.11%	4.59E-05	-0.01%	1.54%	0.434241	0.28%	1.48%	0.764338	chr4	55987799
cg02803139	-0.32%	0.08%	3.98E-05	0.06%	1.75%	0.618689	-0.06%	1.91%	0.728298	chr10	106113391
cg02998936	-0.15%	0.13%	0.236078	-0.51%	1.06%	2.35E-05	-0.26%	1.56%	0.721713	chr17	76338383
cg03060468	-0.02%	0.23%	0.944786	1.11%	1.57%	8.83E-06	-0.45%	1.89%	0.239393	chr5	140683196
cg03260991	-0.54%	0.13%	4.01E-05	0.25%	1.79%	0.288689	-0.13%	1.86%	0.393158	chr19	7539710
cg03261783	0.26%	0.15%	0.074883	0.06%	1.56%	0.519206	-0.16%	1.49%	3.59E-05	chr13	100497453
cg03270969	-0.66%	0.16%	2.58E-05	-0.09%	1.85%	0.903271	-0.07%	1.32%	0.165705	chr2	242813189
cg03542891	0.01%	0.07%	0.923996	0.04%	1.58%	0.403461	-0.49%	1.75%	9.36E-06	chr5	83018357
cg03618918	0.00%	0.11%	0.980359	3.34%	1.48%	7.68E-06	0.34%	1.46%	0.000227	chr1	160865097
cg03691818	-0.04%	0.11%	0.75058	-2.27%	3.63%	2.47E-06	-0.27%	2.61%	0.117835	chr12	53085038
cg03692991	0.14%	0.24%	0.57051	0.39%	0.89%	1.18E-05	0.41%	1.47%	0.556018	chr22	38434974
cg03697766	-3.17%	0.68%	3.42E-06	1.63%	8.17%	0.122026	0.29%	6.98%	0.697128	chr16	54848022
cg03820795	-0.06%	0.05%	0.259366	-0.01%	1.75%	0.766744	-0.13%	1.74%	4.68E-05	chr17	4337140

cg04089434	0.52%	0.13%	4.01E-05	0.42%	1.16%	0.547544	0.07%	1.56%	0.003078	chr10	94516971
cg04752871	-0.59%	0.31%	0.056653	0.87%	1.97%	4.57E-07	0.33%	2.04%	0.299203	chr2	121412432
cg04858776	NA	NA	NA	-2.02%	3.34%	3.57E-06	0.05%	1.98%	0.382235	chr11	59318494
cg04918350	0.44%	0.10%	1.32E-05	-0.07%	1.52%	0.353468	-0.10%	1.56%	0.099502	chr8	134124199
cg04946709	-0.18%	0.14%	0.197612	2.90%	1.61%	4.70E-06	0.15%	1.60%	0.62201	chr16	59789030
cg04989706	-0.04%	0.06%	0.467889	0.04%	1.86%	0.988996	0.09%	1.53%	2.13E-05	chr14	50066350
cg05347215	-0.13%	0.17%	0.443597	0.20%	1.11%	1.27E-05	0.48%	1.24%	0.563788	chr14	67800017
cg05379350	-0.28%	0.22%	0.185935	-0.32%	1.37%	7.96E-06	0.31%	1.39%	0.164225	chr17	27917157
cg05414135	-0.04%	0.23%	0.84767	0.34%	1.76%	0.286769	-1.31%	2.03%	5.38E-06	chr8	56238612
cg05528918	-0.01%	0.14%	0.954917	-0.08%	1.63%	0.598899	-0.01%	1.54%	3.95E-05	chr18	5293319
cg05725804	0.04%	0.07%	0.564449	-0.03%	1.76%	0.371402	-0.39%	2.22%	4.10E-05	chr5	114516219
cg06253734	-0.05%	0.03%	0.143013	-0.01%	1.26%	0.710287	0.07%	1.53%	4.22E-06	chr15	59042325
cg06293745	-0.04%	0.11%	0.696559	0.06%	1.28%	0.107823	-0.09%	1.46%	3.38E-06	chr7	87329407
cg06375652	NA	NA	NA	-0.38%	2.86%	0.01925	-0.18%	2.10%	2.98E-05	chr16	86100423
cg06529894	NA	NA	NA	-0.31%	1.64%	9.33E-06	0.23%	1.66%	0.195002	chr7	76025038
cg06710937	-0.20%	0.18%	0.259766	-1.37%	3.20%	1.84E-05	-0.41%	3.07%	0.040583	chr13	23489940
cg06995408	0.73%	0.17%	2.94E-05	0.25%	1.39%	0.053984	0.50%	1.58%	0.909042	chr2	48977089
cg07116631	0.28%	0.21%	0.184372	0.76%	1.68%	2.12E-05	0.02%	1.22%	0.703596	chr16	3345463
cg07576222	0.08%	0.14%	0.550276	0.18%	1.44%	0.16456	-1.01%	1.40%	1.11E-05	chr15	67357975
cg08406348	0.05%	0.10%	0.619002	-0.06%	1.80%	0.810592	-0.32%	1.62%	2.39E-05	chr2	75700566
cg08961450	0.15%	0.21%	0.464574	-0.78%	1.75%	2.25E-05	0.64%	1.75%	0.182116	chr8	66699054
cg09796146	NA	NA	NA	-0.76%	2.13%	0.678347	-1.46%	1.84%	3.23E-05	chr16	33937240
cg09973676	1.22%	0.29%	3.20E-05	0.50%	4.99%	0.050522	0.11%	4.25%	0.718994	chr8	82006417
cg10074775	0.03%	0.09%	0.751363	0.09%	1.50%	0.784656	-0.51%	1.96%	3.20E-06	chr8	77595027
cg10142520	0.06%	0.20%	0.763548	-0.45%	2.13%	4.43E-05	0.36%	1.58%	0.638851	chr11	65344604
cg10430077	-0.24%	0.33%	0.473784	0.19%	2.01%	1.36E-05	0.21%	2.12%	0.763502	chr5	180648553
cg17238319	NA	NA	NA	0.21%	1.18%	0.961465	-0.75%	1.55%	3.00E-05	chr3	16428391
cg10848373	-0.58%	0.29%	0.045105	-1.40%	2.04%	1.79E-05	-0.22%	2.15%	0.797891	chr21	39047855
cg10881225	0.04%	0.22%	0.838412	0.48%	3.23%	0.903329	2.75%	2.99%	3.95E-05	chr2	9984929

cg11223893	0.01%	0.13%	0.935917	0.20%	1.27%	4.84E-05	0.37%	1.43%	0.690402	chr2	64789303
cg11336539	0.36%	0.16%	0.024085	-0.59%	1.31%	1.64E-05	0.21%	1.44%	0.396738	chr1	28994987
cg11643285	0.03%	0.11%	0.781705	-5.56%	3.73%	7.28E-06	-0.54%	2.20%	0.010997	chr3	16411667
cg11712936	0.06%	0.15%	0.713901	0.79%	1.26%	4.07E-05	0.04%	1.38%	0.393501	chr2	124782117
cg11746684	-0.31%	0.17%	0.072876	-1.47%	2.04%	3.02E-05	0.02%	1.77%	0.81231	chr8	11057265
cg11827082	-0.08%	0.04%	0.070895	0.02%	1.50%	0.835144	0.08%	1.61%	2.20E-05	chr13	50202584
cg11854659	-0.13%	0.09%	0.157219	0.02%	1.67%	0.595663	-0.32%	1.75%	3.28E-05	chr12	113952948
cg12090003	-0.15%	0.12%	0.227628	0.47%	2.47%	2.95E-05	-0.16%	2.47%	0.05132	chr19	17516282
cg12699865	-0.65%	0.13%	7.63E-07	0.21%	0.85%	0.171512	-0.17%	1.12%	0.18146	chr20	32583031
cg13222915	0.01%	0.15%	0.961776	1.22%	0.94%	1.93E-05	0.08%	1.14%	0.377192	chr1	184598594
cg13258831	0.21%	0.14%	0.136942	0.34%	1.96%	0.002771	-0.29%	2.11%	9.09E-06	chr3	11939949
cg13492340	-0.12%	0.15%	0.432585	0.06%	2.05%	0.567754	-0.74%	2.32%	4.08E-05	chr10	15762222
cg14001992	-0.68%	0.16%	1.50E-05	0.17%	1.87%	0.443054	-0.46%	2.08%	0.415067	chr4	154073813
cg15071923	0.48%	0.21%	0.018627	0.09%	2.34%	0.927675	-0.85%	2.51%	2.56E-05	chr12	130623617
cg16142349	-0.03%	0.05%	0.556901	-0.02%	1.23%	0.960833	0.07%	1.07%	2.25E-05	chr6	166911009
cg16254267	-0.77%	0.18%	2.17E-05	-0.40%	1.80%	0.65565	0.15%	2.05%	0.587307	chr1	1073529
cg16416603	-0.11%	0.12%	0.370618	-0.26%	1.40%	1.86E-05	0.29%	1.63%	0.458625	chr20	57593014
cg17238319	NA	NA	NA	-3.94%	1.94%	1.35E-06	0.06%	1.54%	0.4888	chr3	16428391
cg17738863	NA	NA	NA	-0.10%	1.15%	0.177131	-0.25%	1.47%	1.42E-05	chr6	28834489
cg17961932	0.07%	0.10%	0.506519	0.06%	1.72%	0.573849	-0.15%	1.53%	3.25E-05	chr19	54134044
cg18744273	-0.02%	0.14%	0.87694	0.09%	1.89%	0.968975	-0.21%	1.64%	9.65E-06	chr13	112632730
cg20009332	0.29%	0.30%	0.344268	0.52%	1.94%	0.092174	-1.77%	1.89%	7.27E-07	chr8	10489118
cg20064848	0.74%	0.18%	4.90E-05	-0.07%	1.93%	0.488369	-0.39%	1.94%	0.345173	chr1	160037877
cg20301308	0.11%	0.16%	0.481726	-0.35%	1.71%	4.17E-05	-1.08%	2.02%	0.043506	chr1	65534742
cg20444381	-0.06%	0.08%	0.429446	0.12%	0.95%	0.693431	-0.51%	1.24%	4.57E-05	chr8	109456000
cg20484086	0.02%	0.15%	0.881319	-0.67%	1.58%	4.93E-05	0.04%	2.13%	0.804228	chr1	227015727
cg20606555	0.32%	0.21%	0.126941	1.15%	1.74%	3.77E-05	-0.34%	2.04%	0.606901	chr19	52452094
cg20712043	0.60%	0.14%	1.12E-05	-0.03%	1.62%	0.567598	0.36%	1.36%	0.378657	chr5	16392700
cg20995573	-0.10%	0.21%	0.641493	0.33%	2.90%	0.997782	-1.10%	3.09%	3.65E-05	chr22	51038379

cg21142743	0.15%	0.20%	0.430637	0.27%	1.28%	1.26E-06	0.04%	1.77%	0.93481	chr1	228272495
cg21151899	NA	NA	NA	0.78%	1.57%	2.98E-06	0.54%	1.36%	0.020499	chr22	42337657
cg21216130	-0.03%	0.25%	0.919977	1.54%	2.83%	2.99E-06	0.72%	2.59%	0.494365	chr20	170511
cg21368354	-0.07%	0.06%	0.217147	-0.02%	2.05%	0.343779	-0.42%	2.27%	2.11E-06	chr13	111267856
cg21578543	0.09%	0.11%	0.452344	-0.10%	1.91%	0.414955	-0.14%	1.70%	4.67E-05	chr4	155312795
cg21926402	0.04%	0.10%	0.684326	-0.04%	4.33%	0.853989	-0.63%	5.24%	2.92E-05	chr3	148804275
cg21986027	0.74%	0.17%	1.35E-05	0.06%	3.06%	0.180709	-0.02%	2.72%	0.996688	chr6	169238138
cg22082046	0.06%	0.21%	0.79125	-0.88%	1.74%	2.23E-05	-0.46%	2.02%	0.184891	chr9	19934447
cg22638542	-0.04%	0.09%	0.641233	0.04%	1.47%	0.512473	0.48%	1.93%	4.65E-05	chr3	42624573
cg22894517	0.02%	0.12%	0.857639	0.29%	0.80%	2.94E-06	-0.12%	1.01%	0.488227	chr11	124733038
cg23052793	-0.37%	0.14%	0.008294	0.06%	1.23%	0.089738	0.11%	1.22%	8.45E-06	chr1	84547955
cg23167307	0.00%	0.12%	0.981477	-0.08%	1.07%	0.552002	-0.65%	1.12%	1.46E-06	chr17	36981148
cg23256480	0.19%	0.05%	3.44E-05	-0.01%	1.38%	0.837246	0.03%	1.58%	0.512898	chr14	93252030
cg23308184	0.12%	0.11%	0.274312	0.02%	1.78%	0.837778	-0.40%	2.02%	7.55E-06	chr10	132977221
cg23337116	-0.02%	0.04%	0.560326	-0.01%	1.07%	0.216923	-0.13%	1.51%	3.45E-05	chr4	53728510
cg23482397	0.00%	0.06%	0.969035	-0.06%	0.99%	1.58E-05	-0.04%	1.06%	0.64058	chr18	53257226
cg23501767	-0.01%	0.03%	0.825484	0.04%	0.98%	1.51E-05	-0.06%	1.78%	0.160036	chr5	133340570
cg24168538	NA	NA	NA	1.91%	4.19%	3.04E-05	-0.21%	3.43%	0.790411	chr4	35527016
cg24213719	-0.01%	0.05%	0.765948	0.02%	1.73%	0.963285	-0.36%	3.02%	4.15E-05	chr18	60263646
cg25203085	-0.59%	0.13%	7.80E-06	0.06%	2.34%	0.344623	0.22%	2.13%	0.156841	chr7	887678
cg25304146	NA	NA	NA	2.23%	0.91%	1.02E-05	-0.01%	1.14%	0.917899	ch18	30092971
cg25401210	-0.07%	0.07%	0.307487	-0.16%	1.77%	2.40E-05	0.05%	1.12%	0.356796	chr7	944776
cg25485956	-0.11%	0.03%	3.27E-05	0.00%	0.87%	0.053326	0.00%	0.86%	0.183876	chr10	81146099
cg26033520	0.07%	0.17%	0.702252	-1.61%	2.53%	2.07E-06	0.46%	2.34%	0.61209	chr10	74004071
cg26121782	-0.05%	0.06%	0.363083	0.08%	3.48%	0.10161	0.60%	4.69%	4.05E-05	chr11	8284312
cg26166854	0.17%	0.27%	0.515667	0.32%	2.33%	1.81E-05	1.06%	2.66%	0.265365	chr6	6614447
cg26273417	0.03%	0.11%	0.773365	0.51%	2.00%	3.08E-05	-0.09%	2.30%	0.19174	chr13	107146164
cg26337020	0.01%	0.02%	0.699724	0.03%	0.82%	3.01E-05	-0.01%	0.89%	0.819508	chr5	100239022
cg26355737	NA	NA	NA	2.76%	2.63%	2.90E-05	0.19%	1.99%	0.210928	chr13	114292172

cg26944434	-0.04%	0.06%	0.560297	0.03%	1.60%	0.437694	-0.27%	1.91%	1.82E-05	chr10	112064524

Table S6. 45 ASD-associated DMPs were identified at the "discovery" P-value threshold (P < 5 x 10^{-5}) in our EWAS meta-analysis. Genomic locations are based on hg19.

Probe ID	Minerva			SEED			Simons			Meta-analysis P value	N studies	Chr	Pos
	Mean Diff	SE	P-value	Mean Diff	SE	P value	Mean Diff	SE	P value				
cg00692367	0.80%	0.18%	1.68E-05	0.28%	1.98%	0.3858205	-0.32%	1.70%	0.0230783	2.09E-05	3	chr1	211665511
cg01716316	0.06%	0.04%	0.1392718	-0.09%	1.46%	0.0013335	-0.13%	1.65%	0.000637	1.71E-05	3	chr17	40897182
cg01722932	-0.54%	0.18%	0.0022416	-0.15%	1.19%	0.0017174	0.51%	1.30%	0.0045474	3.12E-06	3	chr11	92702653
cg01798266	0.05%	0.13%	0.7139732	-0.43%	1.17%	2.39E-05	-0.13%	1.47%	0.0129828	2.96E-05	3	chr1	153320414
cg03618918	0.00%	0.11%	0.9803587	3.34%	1.48%	7.68E-06	0.34%	1.46%	0.0002273	3.85E-07	3	chr1	160865097
cg03691818	-0.04%	0.11%	0.7505796	-2.27%	3.63%	2.47E-06	-0.27%	2.61%	0.1178351	2.93E-05	3	chr12	53085038
cg03697766	-3.17%	0.68%	3.42E-06	1.63%	8.17%	0.1220258	0.29%	6.98%	0.6971282	3.76E-05	3	chr16	54848022
cg03933495	-0.54%	0.18%	0.002669	0.68%	1.27%	0.0018707	-0.52%	1.35%	0.0436744	2.92E-05	3	chr16	3493614
cg04089434	0.52%	0.13%	4.01E-05	0.42%	1.16%	0.5475445	0.07%	1.56%	0.003078	1.04E-05	3	chr10	94516971
cg04752871	-0.59%	0.31%	0.0566526	0.87%	1.97%	4.57E-07	0.33%	2.04%	0.2992029	1.50E-06	3	chr2	121412432
cg05379350	-0.28%	0.22%	0.1859355	-0.32%	1.37%	7.96E-06	0.31%	1.39%	0.1642249	3.21E-05	3	chr17	27917157
cg06293745	-0.04%	0.11%	0.6965595	0.06%	1.28%	0.1078233	-0.09%	1.46%	3.38E-06	3.34E-05	3	chr7	87329407
cg06710937	-0.20%	0.18%	0.2597656	-1.37%	3.20%	1.84E-05	-0.41%	3.07%	0.0405828	2.64E-05	3	chr13	23489940
cg07105285	-0.65%	0.28%	0.019963	-0.21%	3.37%	0.0095307	1.22%	3.38%	0.0013282	3.32E-05	3	chr11	92702663
cg07116947	-0.89%	0.23%	8.03E-05	0.64%	1.49%	0.0001224	0.35%	1.73%	0.0769941	1.84E-07	3	chr17	1933006
cg08217285	-0.23%	0.26%	0.3886416	-0.09%	1.55%	0.0006928	0.71%	1.67%	0.0007565	2.75E-05	3	chr17	27917879
cg09937849	0.75%	0.25%	0.0032092	0.62%	1.87%	0.0008199	0.37%	2.11%	0.0454524	1.72E-05	3	chr16	318717
cg09962502	0.01%	0.05%	0.880838	-0.05%	0.97%	0.0002396	-0.08%	0.88%	0.0015908	4.26E-05	3	chr2	96971189
cg11336539	0.36%	0.16%	0.0240851	-0.59%	1.31%	1.64E-05	0.21%	1.44%	0.3967376	2.19E-05	3	chr1	28994987
cg11643285	0.03%	0.11%	0.7817052	-5.56%	3.73%	7.28E-06	-0.54%	2.20%	0.0109974	9.71E-06	3	chr3	16411667
cg12090003	-0.15%	0.12%	0.2276278	0.47%	2.47%	2.95E-05	-0.16%	2.47%	0.0513197	4.37E-05	3	chr19	17516282
cg12159190	0.14%	0.04%	0.0002512	0.03%	0.91%	0.0011738	-0.02%	0.88%	0.3078814	1.35E-05	3	chr8	80679978
cg12699865	-0.65%	0.13%	7.63E-07	0.21%	0.85%	0.1715117	-0.17%	1.12%	0.1814597	4.10E-06	3	chr20	32583031
cg13258831	0.21%	0.14%	0.1369418	0.34%	1.96%	0.0027714	-0.29%	2.11%	9.09E-06	7.26E-07	3	chr3	11939949

cg15154047	-0.06%	0.19%	0.7594188	1.34%	1.44%	5.19E-05	0.57%	1.23%	0.0084075	4.21E-05	3	chr8	143210309
cg16025847	-0.14%	0.06%	0.0188738	-0.08%	1.69%	0.0826959	-0.37%	2.32%	8.81E-05	1.95E-05	3	chr12	129338484
cg16867777	0.36%	0.10%	0.0006024	0.00%	1.33%	0.9787983	0.03%	1.30%	0.0001729	1.50E-05	3	chr21	32932073
cg20009332	0.29%	0.30%	0.3442677	0.52%	1.94%	0.0921743	-1.77%	1.89%	7.27E-07	4.00E-06	3	chr8	10489118
cg20236995	-0.55%	0.16%	0.0004842	-0.63%	0.82%	0.000627	-0.20%	0.98%	0.286996	1.30E-05	3	chr1	3071963
cg20580913	-0.72%	0.23%	0.002299	-0.09%	4.39%	0.5533035	0.94%	3.89%	7.95E-05	1.49E-05	3	chr6	1599288
cg21368354	-0.07%	0.06%	0.2171473	-0.02%	2.05%	0.3437788	-0.42%	2.27%	2.11E-06	2.20E-05	3	chr13	111267856
cg22761670	0.46%	0.13%	0.0004195	0.48%	3.00%	0.0230648	0.21%	2.84%	0.0253839	3.24E-05	3	chr17	74268201
cg22788646	-0.30%	0.12%	0.0128503	-0.25%	1.99%	0.0015574	0.30%	2.09%	0.0135527	3.54E-05	3	chr12	132512003
cg23052793	-0.37%	0.14%	0.0082943	0.06%	1.23%	0.089738	0.11%	1.22%	8.45E-06	1.25E-06	3	chr1	84547955
cg23193153	-0.21%	0.06%	0.0003114	-0.13%	1.53%	0.0007322	-0.04%	1.78%	0.496042	1.64E-05	3	chr6	34192178
cg25485956	-0.11%	0.03%	3.27E-05	0.00%	0.87%	0.0533258	0.00%	0.86%	0.1838762	4.10E-05	3	chr10	81146099
cg02606018	NA	NA	NA	1.80%	0.87%	7.55E-07	-0.10%	0.87%	0.3625623	4.41E-06	2	chr12	10658281
cg03731974	NA	NA	NA	0.09%	2.58%	0.0001876	0.08%	1.84%	0.001597	4.80E-06	2	chr16	86531598
cg04858776	NA	NA	NA	-2.02%	3.34%	3.57E-06	0.05%	1.98%	0.3822349	1.98E-05	2	chr11	59318494
cg06152526	NA	NA	NA	1.83%	1.54%	6.13E-05	0.60%	1.44%	0.000648	7.16E-07	2	chr16	57290525
cg06375652	NA	NA	NA	-0.38%	2.86%	0.0192505	-0.18%	2.10%	2.98E-05	8.83E-06	2	chr16	86100423
cg06529894	NA	NA	NA	-0.31%	1.64%	9.33E-06	0.23%	1.66%	0.1950018	2.59E-05	2	chr7	76025038
cg17238319	NA	NA	NA	-3.94%	1.94%	1.35E-06	0.06%	1.54%	0.4888002	1.00E-05	2	chr3	16428391
cg17738863	NA	NA	NA	-0.10%	1.15%	0.1771313	-0.25%	1.47%	1.42E-05	3.50E-05	2	chr6	28834489
cg21151899	NA	NA	NA	0.78%	1.57%	2.98E-06	0.54%	1.36%	0.020499	1.07E-06	2	chr22	42337657

Table S7. All polygenic risk scores based on P-values < 1 from the PGC-AUT GWAS significantly predicted autism status in the MINERvA cohort.

P_T		PRS against ASD status		
		Estimate	P-value	R2
S1	$5x10^{-8}$	1.077202049	0.243967383	0.08%
S2	$1x10^{-6}$	0.658597093	0.013704071	0.35%
S3	$1x10^{-4}$	0.24409224	0.000599044	0.69%
S4	0.001	0.156618181	2.44E-05	1.05%
S5	0.01	0.102263694	4.16E-08	1.80%
S6	0.05	0.079691112	5.08E-12	2.91%
S7	0.1	0.069335858	9.49E-13	3.12%
S8	0.2	0.057158699	7.76E-12	2.86%
S9	0.5	0.049941136	1.14E-11	2.81%
S10	1	0.048193781	2.40E-11	2.71%

Table S8. 49 DMPs were associated with ASD PRS at "discovery" P-value threshold ($P < 5 \times 10^{-5}$). Genomic locations are based on hg19.

See **Additional File 3** for table.

Table S9. ASD PRS EWAS of cg02771117 iteratively adding PRS SNPs (ordered by significance in ASD GWAS) into the model.

nGWAS SNPs added to regression model	PRS EWAS			nGWAS SNPs significant in PRS model	Significant GWAS SNPs
	RegCoeff	SE	P-value		
0	-0.21%	0.04%	3.14E-08	0	
1	-0.13%	0.04%	0.000256109	1	rs10099100
2	-0.09%	0.04%	0.014457347	2	rs10099100 ; rs2736342
3	-0.08%	0.04%	0.020616094	3	rs10099100 ; rs2736342 ; rs7000276
4	-0.07%	0.04%	0.051798387	4	rs10099100 ; rs2736342 ; rs7000276 ; rs4240654
5	-0.06%	0.04%	0.092042966	3	rs10099100 ; rs2736342 ; rs7000276
6	-0.07%	0.04%	0.07932783	3	rs10099100 ; rs2736342 ; rs13264212
7	-0.07%	0.04%	0.072944444	3	rs10099100 ; rs2736342 ; rs4240654
8	-0.06%	0.04%	0.113003576	3	rs10099100 ; rs2736342 ; rs1962656
9	-0.07%	0.04%	0.08448709	3	rs10099100 ; rs2736342 ; rs1962656
10	-0.08%	0.04%	0.068911894	3	rs10099100 ; rs2736342 ; rs1962656
11	-0.08%	0.04%	0.070110576	3	rs10099100 ; rs2736342 ; rs1962656
12	-0.07%	0.04%	0.093849792	2	rs2736342 ; rs1962656
13	-0.08%	0.04%	0.079300615	2	rs2736342 ; rs2736372
14	-0.07%	0.04%	0.091784474	2	rs2736342 ; rs2736372
15	-0.07%	0.04%	0.104285362	2	rs2736342 ; rs2736372
16	-0.07%	0.04%	0.119649518	4	rs10099100 ; rs2736342 ; rs2736372 ; rs17723593
17	-0.06%	0.04%	0.179799809	4	rs2736342 ; rs4841453 ; rs2736372 ; rs17723593
18	-0.06%	0.05%	0.18028655	3	rs2736342 ; rs2736372 ; rs17723593
19	-0.05%	0.05%	0.310655962	4	rs2736342 ; rs2736372 ; rs80084058 ; rs17723593
20	-0.04%	0.05%	0.342654831	3	rs2736342 ; rs4841453 ; rs80084058
21	-0.04%	0.05%	0.432902668	4	rs10099100 ; rs2736342 ; rs4841453 ; rs80084058
22	-0.04%	0.05%	0.386086461	5	rs10099100 ; rs2736342 ; rs4841453 ; rs80084058 ; rs6994363
23	-0.05%	0.05%	0.343314664	5	rs10099100 ; rs2736342 ; rs4841453 ; rs80084058 ; rs6994363

24	-0.03%	0.04%	0.557619251	6	rs10099100 ; rs1962656 ; rs4841453 ; rs2736372 ; rs6994363 ; rs66600736
25	-0.04%	0.05%	0.362964924	5	rs10099100 ; rs1962656 ; rs4841453 ; rs2736372 ; rs66600736
26	-0.05%	0.05%	0.270969593	6	rs10099100 ; rs1962656 ; rs4841453 ; rs2736372 ; rs66600736 ; rs55661112
27	-0.05%	0.05%	0.275776686	4	rs1962656 ; rs4841453 ; rs2736372 ; rs66600736
28	-0.06%	0.05%	0.257870861	5	rs10099100 ; rs1962656 ; rs4841453 ; rs2736372 ; rs66600736
29	-0.05%	0.05%	0.305635393	4	rs1962656 ; rs4841453 ; rs2736372 ; rs66600736
30	-0.03%	0.05%	0.49713562	4	rs1962656 ; rs4841453 ; rs66600736 ; rs55661112
31	-0.06%	0.05%	0.279781894	4	rs1962656 ; rs4841453 ; rs66600736 ; rs55661112
32	-0.06%	0.05%	0.269514236	4	rs1962656 ; rs4841453 ; rs66600736 ; rs55661112
33	-0.04%	0.05%	0.385112439	8	rs2736342 ; rs1962656 ; rs4841453 ; rs12681639 ; rs2736326 ; rs66600736 ; rs55661112 ; rs11250139

Table S10. ASD PRS EWAS of cg27411982 iteratively adding PRS SNPs (ordered by significance in ASD GWAS) into the model.

nGWAS SNPs added to regression model	PRS EWAS			nGWAS SNPs significant in PRS model	Significant GWAS SNPs
	RegCoeff	SE	P-value		
0	-0.07%	0.01%	8.38E-08	0	
1	-0.05%	0.01%	1.95E-05	1	rs10099100
2	-0.04%	0.01%	0.001574671	1	rs2736342
3	-0.04%	0.01%	0.002808104	2	rs2736342 ; rs7000276
4	-0.03%	0.01%	0.005077792	2	rs2736342 ; rs7000276
5	-0.04%	0.01%	0.002051182	2	rs2736342 ; rs7000276
6	-0.04%	0.01%	0.002521504	2	rs2736342 ; rs7000276
7	-0.04%	0.01%	0.002389964	2	rs2736342 ; rs7000276
8	-0.04%	0.01%	0.002278556	2	rs2736342 ; rs7000276
9	-0.04%	0.01%	0.002454498	2	rs2736342 ; rs7000276
10	-0.04%	0.01%	0.002704491	3	rs2736342 ; rs7000276 ; rs4841406
11	-0.04%	0.01%	0.002675687	3	rs2736342 ; rs7000276 ; rs4841406
12	-0.03%	0.01%	0.025065592	4	rs2736342 ; rs7000276 ; rs4841406 ; rs12681639
13	-0.03%	0.01%	0.020136528	5	rs2736342 ; rs1962656 ; rs4841406 ; rs12681639 ; rs2736372
14	-0.04%	0.01%	0.008400483	5	rs2736342 ; rs1962656 ; rs4841406 ; rs12681639 ; rs2736372
15	-0.04%	0.01%	0.009221259	6	rs2736342 ; rs58075522 ; rs1962656 ; rs4841406 ; rs12681639 ; rs2736372
16	-0.04%	0.01%	0.007776977	5	rs2736342 ; rs1962656 ; rs4841406 ; rs12681639 ; rs2736372
17	-0.04%	0.01%	0.009767081	6	rs2736342 ; rs7000276 ; rs1962656 ; rs4841406 ; rs12681639 ; rs2736372
18	-0.04%	0.01%	0.009496432	6	rs2736342 ; rs7000276 ; rs1962656 ; rs4841406 ; rs12681639 ; rs2736372
19	-0.03%	0.01%	0.012735102	3	rs4841406 ; rs12681639 ; rs7018397
20	-0.03%	0.01%	0.038148299	5	rs7834323 ; rs4841406 ; rs12681639 ; rs7018397 ; rs10095927

21	-0.04%	0.01%	0.017011652	5	rs7834323 ; rs4841406 ; rs12681639 ; rs7018397 ; rs10095927
22	-0.04%	0.01%	0.012741299	5	rs13264212 ; rs7834323 ; rs12681639 ; rs7018397 ; rs10095927
23	-0.04%	0.02%	0.009159004	2	rs12681639 ; rs7018397
24	-0.04%	0.02%	0.010227827	2	rs12681639 ; rs7018397
25	-0.05%	0.02%	0.006028348	4	rs7834323 ; rs1962656 ; rs12681639 ; rs7018397
26	-0.04%	0.02%	0.008310536	4	rs7834323 ; rs1962656 ; rs12681639 ; rs7018397
27	-0.05%	0.02%	0.008001908	3	rs1962656 ; rs12681639 ; rs7018397
28	-0.04%	0.02%	0.011030205	3	rs1962656 ; rs12681639 ; rs7018397
29	-0.05%	0.02%	0.007719218	3	rs1962656 ; rs12681639 ; rs7018397
30	-0.05%	0.02%	0.0068404	4	rs7834323 ; rs1962656 ; rs12681639 ; rs7018397
31	-0.04%	0.02%	0.015376559	2	rs12681639 ; rs7018397
32	-0.04%	0.02%	0.016834492	4	rs12681639 ; rs7018397 ; rs10095927 ; rs2409703
33	-0.04%	0.02%	0.022877821	5	rs7834323 ; rs12681639 ; rs7018397 ; rs10095927 ; rs2409703

Table S11. Bayesian co-localization results for DNA methylation sites and ASD. Genomic locations are based on hg19.

See **Additional File 3** for table.
